# Single‐Cell Sequencing Reveals That CD4 ^+^ T Cells Eliminate Senescent Prostate Epithelium to Delay Progression of Benign Prostatic Hyperplasia

**DOI:** 10.1111/acel.70180

**Published:** 2025-07-27

**Authors:** Zheng Li, Xiaofei Wang, Zhifu Liu, Senmao Li, Zhenan Zhang, Chenchen Huang, Yixiao Liu, Xingxing Tang, Jiaen Zhang, Peimin Zhou, Ying Gan, Yu Fan, Yisen Meng, Kaiwei Yang, Shuai Hu, Qian Zhang, Wei Yu

**Affiliations:** ^1^ Department of Urology, Peking University First Hospital, Institute of Urology, Peking University, Beijing Key Laboratory of Urogenital Diseases (Male), molecular Diagnosis and Treatment Center National Research Center for Genitourinary Oncology Beijing China; ^2^ Xuanwu Hospital Capital Medical University Beijing China; ^3^ Department of Urology, Hunan Provincial People's Hospital The First Affiliated Hospital of Hunan Normal University Changsha China; ^4^ Department of Urology The Second Affiliated Hospital of Kunming Medical University Kunming China; ^5^ Department of Urology, Beijing Shijitan Hospital Capital Medical University Beijing China

**Keywords:** BPH, CD4+ CTL, cellular senescence, CXCL13, single‐cell, Treg

## Abstract

Benign prostatic hyperplasia (BPH) is an age‐related condition characterized by progressive prostate enlargement driven in part by the accumulation of senescent epithelial cells and their pro‐inflammatory secretome. Using human single‐cell RNA sequencing and laser capture microdissection, we identified C‐X‐C Motif Chemokine Ligand 13 (CXCL13) as a key chemokine secreted by senescent prostate epithelial cells. CXCL13 recruits CD4^+^ T cells via the C‐X‐C Chemokine Receptor Type 5 (CXCR5) receptor, facilitating immune recognition through human leukocyte antigen–DR isotype (HLA‐DR) and promoting senescent cell clearance. Functional assays revealed that CD4^+^ cytotoxic T lymphocytes (CTLs) mediate this clearance, while regulatory T cells (Tregs) suppress it, forming a functional dichotomy. Immunohistochemistry, transwell migration, and co‐culture assays confirmed this CXCL13–CXCR5–HLA‐DR axis. In a testosterone‐induced BPH mouse model, CXCL13 treatment enhanced CD4^+^ T cell infiltration and reduced epithelial senescence, while CD4^+^ T cell depletion reversed these effects. Single‐cell transcriptomics in mice further validated increased CXCL13 expression and CD4^+^ T cell engagement. These findings uncover a critical immune surveillance mechanism in BPH and suggest that targeting the CXCL13–CD4^+^ T cell axis may offer a novel therapeutic strategy for age‐related prostate enlargement.

## Graphical abstract

Senescent BPH epithelial cells secrete CXCL13 to recruit CD4^+^ T cells via CXCR5. Cytotoxic CD4^+^ T cells eliminate senescent cells through MHC‐II–dependent recognition. Tregs suppress, while B cells show minimal effect. This immune axis modulates senescent cell clearance and may influence BPH progression.

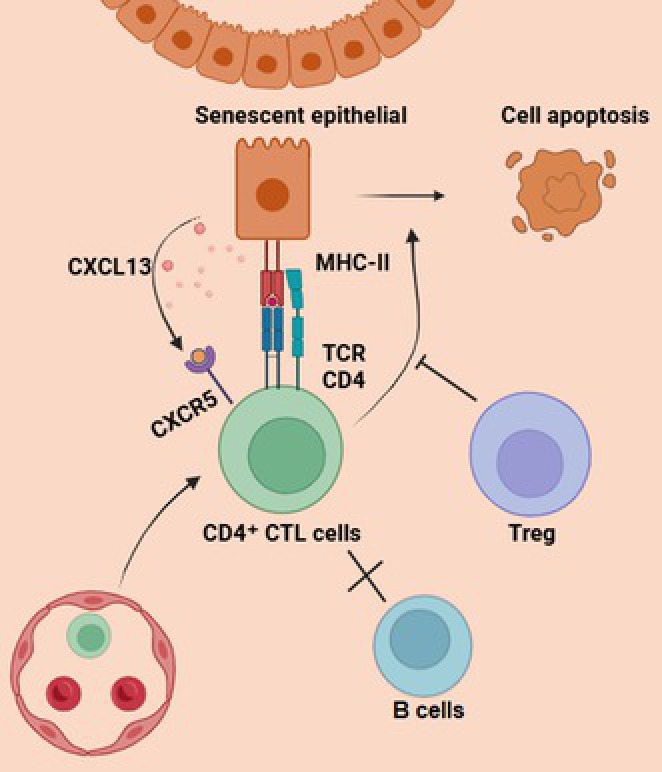

## 1 Introduction

Benign prostatic hyperplasia (BPH) is highly prevalent among aging men, affecting over 50% of those aged 60 and above (Chughtai et al. 2016). This progressive enlargement of the prostate leads to urinary obstruction and a considerable decline in quality of life. Reflecting its impact, BPH had the highest age‐standardized incidence rate (ASIR) among urologic diseases in 2021, reaching 5531.88 cases per 100,000 persons (Zi et al. 2024). Central to BPH pathophysiology is the accumulation of senescent cells in the prostate epithelium and stroma (Castro et al. 2003; Choi et al. 2000; Fiard et al. 2021). Although non‐proliferative, these cells remain metabolically active and secrete a suite of pro‐inflammatory and tissue‐remodeling molecules known collectively as the senescence‐associated secretory phenotype (SASP) (Vital et al. 2014).

Key SASP factors—including interleukin‐1alpha (IL‐1α), interleukin‐6 (IL‐6), interleukin‐8 (IL‐8), and matrix metalloproteinases (MMPs)—are instrumental in driving chronic inflammation, fibrosis, and the proliferation of epithelial and stromal cells, thereby exacerbating prostate enlargement and accelerating BPH progression (Castro et al. 2004; Jiang et al. 2019; Panchanathan et al. 2021; Untergasser et al. 2005). This SASP milieu establishes a pro‐inflammatory microenvironment that actively recruits and activates immune cells, leading to persistent tissue remodeling and fibrosis, hallmarks of BPH‐associated hyperplasia (Lasry and Ben‐Neriah 2015).

The immune system plays an essential role in maintaining tissue homeostasis, particularly in response to cellular senescence. Various immune cells, such as macrophages, natural killer (NK) cells, and T lymphocytes, are critical in recognizing and clearing senescent cells (Deng et al. 2024; He and Sharpless 2017; Marin et al. 2023; Zhao et al. 2022; Zhu et al. 2023). Macrophages engulf senescent cells, while NK cells exert cytotoxic effects to eliminate them (Sroka et al. 2023; Wang et al. 2022). Among T lymphocytes, CD4+ T cells are pivotal, orchestrating immune responses against senescent cells through chemokine interactions and SASP signaling (Hasegawa et al. 2023). However, the specific mechanisms by which CD4+ T cells mediate the clearance of senescent cells in BPH remain incompletely understood.

Recent advancements in single‐cell transcriptomics have significantly deepened our understanding of cellular senescence across various biological systems (Gurkar et al. 2023; Moiseeva, Cisneros, Sica, et al. 2023; Tao et al. 2024). This technology enables high‐resolution profiling of individual cells, identifying senescent cells via markers such as p16^INK4a, p21^CIP1, and key SASP components (Gratzke et al. 2015; Tan et al. 2024). While single‐cell sequencing has elucidated complex interactions between senescent and immune cells in diverse pathological contexts, its application to BPH is only beginning to unfold.

In this study, we investigate how CD4^+^ T cells recognize and eliminate senescent epithelial cells (ECs) in BPH prostate tissues and how this immune surveillance influences disease progression. We propose that senescent cells secrete CXCL13 to recruit CD4^+^ T cells via CXCR5, enabling HLA‐DR‐dependent antigen recognition and clearance. A distinct subset of cytotoxic CD4^+^ T cells (CD4^+^ CTLs) emerged as key effectors inducing apoptosis in senescent cells, while Tregs suppressed this process and B cells played a minimal role.

Using single‐cell RNA sequencing (scRNA‐Seq) and laser capture microdissection (LCM), we mapped the immune microenvironment of senescent regions and identified key CD4^+^ T cell interactions. Findings were validated by in vitro co‐culture and chemotaxis assays, confirming the roles of CXCL13–CXCR5 signaling and HLA‐DR‐mediated antigen presentation in CD4^+^ T cell recruitment and cytotoxicity. In vivo models of BPH and epithelial senescence further demonstrated that CD4^+^ T cells, regulated by CXCL13, restrain hyperplasia. These results suggest that enhancing CD4^+^ CTL activity or relieving Treg suppression via the CXCL13–CXCR5 axis may offer a therapeutic strategy for targeting senescent cells in BPH.

## 2 Methods

### 2.1 Patient Samples

Prostate tissues were obtained from patients with BPH undergoing transurethral resection of the prostate (TURP) for lower urinary tract symptoms. Exclusion criteria included prior prostate cancer, confirmed prostatitis, long‐term catheterization, or previous use of α1‐blockers or 5α‐reductase inhibitors.

SA‐β‐gal staining identified senescent and non‐senescent epithelial regions in samples from three patients, yielding three paired samples. An additional 28 BPH tissues were used for immunohistochemistry. All procedures followed ethical guidelines approved by the Institutional Review Board of Peking University First Hospital. Clinical details are in Table S1.

Six single‐cell RNA‐seq datasets (GSE226237), meeting the same inclusion criteria, were retrieved from the GEO database.

### 2.2 scRNA‐Seq and Analysis

scRNA‐seq was used to profile BPH in both human and mouse models. Six human BPH samples (GSE226237) and two from a control and testosterone‐induced BPH mouse model (GSM6547515, GSM6547516) were obtained from the GEO database. Raw data were processed using Seurat v5.0.1 in R (Butler et al. 2018; Satija et al. 2015). Genes expressed in fewer than three cells were excluded, and cells with < 200 or > 2500 detected genes or > 5% mitochondrial content were filtered out. Data normalization was performed using NormalizeData(), and 2000 highly variable genes were identified with FindVariableFeatures(method = “vst”).

Each sample was preprocessed individually, followed by anchor‐based integration using FindIntegrationAnchors(dims = 1:30) and IntegrateData(dims = 1:30). The assay was set to “integrated” for downstream analysis. The dataset was scaled with ScaleData() and subjected to PCA (npcs = 30). Clustering was performed using the first 20 PCs (dims = 1:20) via FindNeighbors() and FindClusters(resolution = 0.5). UMAP (RunUMAP()) was used for dimensionality reduction and visualization. Cell types were annotated based on canonical marker genes, detailed in Table S2.

### 2.3 Violin Plot Visualization

Gene expression distributions were visualized using Seurat's VlnPlot() function, grouped by annotated cell types. Point size (pt.size) was adjusted for clarity. Plots were further refined with ggplot2 for consistent axis labels, font sizes, and color schemes.

### 2.4 Gene Set Variation Analysis (GSVA) and Gene Set Enrichment Analysis (GSEA)

Gene set enrichment analysis (GSEA) was performed using the fgsea package v1.16 (Guo and Zhu 2024) on ranked gene lists from differential expression analysis (*p* < 0.05, log_2_FC threshold), with gene sets from MSigDB v7.5.1 (Hallmark, KEGG, Reactome) (Liberzon et al. 2011; Subramanian et al. 2005). GSVA was conducted using the GSVA package v1.40 (Hänzelmann et al. 2013) to assess senescence‐related pathway activity, also based on MSigDB. GSVA scores were projected onto UMAPs to visualize cell‐level pathway enrichment.

### 2.5 Differential Gene Expression (DEG) Analysis

To avoid inflated significance from treating individual cells as independent, differential gene expression (DEG) analysis was performed using a pseudobulk strategy with Seurat's AggregateExpression() function, aggregating counts at the sample level for statistical robustness.

For human scRNA‐seq data (*n* = 6 BPH patients, GSE226237), ECs were extracted per patient and aggregated by sample ID. DEG analysis was conducted using the limma‐voom pipeline (Law et al. 2014; Ritchie et al. 2015), modeling mean–variance relationships and applying empirical Bayes moderation for stable variance estimates.

For mouse data (1 BPH vs. 1 control), ECs were aggregated by condition. Though lacking biological replicates, pseudobulk analysis reduced bias from unequal cell numbers.

For paired senescent vs. non‐senescent regions from a single BPH sample (identified by SA‐β‐gal staining), DEGs were identified using Seurat's FindMarkers() function. Results from this single‐sample comparison were interpreted cautiously due to the absence of replicates.

DEG results were visualized with EnhancedVolcano, highlighting senescence‐related genes.

### 2.6 Cell–Cell Interaction Analysis Using CellChat

#### 2.6.1 Human Data (*n* = 6 Samples)

Cell–cell communication in human BPH tissues was analyzed using the CellChat package v1.1.0 (Jin et al. 2021) in R. Single‐cell transcriptomes from six patients were individually processed and integrated via Seurat before interaction inference. CellChat identified signaling networks based on known ligand–receptor pairs, with a focus on CXCL signaling from senescent vs. non‐senescent ECs to CD4^+^ T cells.

Interaction strength and count were quantified per sample. Sample‐wise differences (Δ = senescent—non‐senescent) in CD4^+^‐directed signaling were assessed using paired *t*‐tests and Cohen's *d* for effect size. Network visualizations (circle plots, chord diagrams, hierarchical layouts) were generated using CellChat functions (netVisual_circle(), netVisual_aggregate()), and high‐resolution TIFFs were exported.

#### 2.6.2 Mouse Data (*n* = 2 Samples)

scRNA‐seq data from one BPH and one control mouse were analyzed with Seurat and CellChat, focusing on epithelial‐to‐CD4^+^ T cell CXCL signaling. Due to limited sample size, statistical testing was not performed; interactions were interpreted descriptively. Visualizations were generated as in the human data using CellChat's standard plotting functions.

### 2.7 Senescence‐Associated β‐Galactosidase Staining

Fresh‐frozen human BPH tissues were cryosectioned at 8–10 μm and mounted on glass slides. SA‐β‐gal staining was performed using a commercial kit (C0602, Beyotime, China) per manufacturer's protocol. Sections were fixed for 10–15 min at room temperature, washed, and incubated overnight at 37°C (no CO_2_) in X‐gal staining solution (pH 6.0). Blue staining indicated SA‐β‐gal^+^ senescent cells, which were imaged by brightfield microscopy.

### 2.8 LCM

LCM was used to isolate senescent (SA‐β‐Gal^+^) and non‐senescent ECs from serial sections of fresh‐frozen BPH tissue. Dissection followed the manufacturer's protocol using the MMI CellCut Plus system (Gene Company Limited, Hong Kong), ensuring precise capture and RNA quality for transcriptomic analysis.

### 2.9 Bulk RNA‐Seq and Functional Enrichment Analysis of Microdissected Epithelial Cells

Total RNA was extracted from laser‐microdissected senescent and non‐senescent epithelial regions of BPH tissues. RNA quality was assessed using the Agilent 2100 Bioanalyzer. Libraries were prepared from ≥ 1 μg RNA via poly(A) + selection, fragmentation, cDNA synthesis, end repair, adapter ligation, and PCR amplification. Library quality was confirmed by Qubit, size distribution, and qPCR. Sequencing was performed on an Illumina platform (150 bp paired‐end reads).

Reads were trimmed and aligned to the human genome (GRCh38) using HISAT2; gene counts were obtained with featureCounts. Differential expression analysis was performed using limma on pseudobulk matrices. Ranked gene lists (logFC) were input into GSEA via GSEA() in clusterProfiler, with gene sets from MSigDB via msigdbr.

For KEGG pathway analysis, gene symbols were converted to Entrez IDs using bitr(), followed by enrichment via enrichKEGG(). Pathways related to senescence and CD4 ^+^ T cell chemotaxis/activation were visualized using GSEA enrichment plots and ggplot2‐generated bubble plots, with gene ratio (*x*‐axis), pathway name (*y*‐axis), and –log_10_(*p*‐value) as color scale.

### 2.10 Venn Diagram and ADEIP (Age‐Dependent Expression and Immune Profile Database) Database Comparison

A Venn diagram was used to compare DEGs from senescent ECs with the ADEIP database (Liu et al. 2021), highlighting shared genes such as CXCL13 to identify those linked to both prostate aging and BPH pathogenesis.

### 2.11 Immunohistochemical Staining

Immunohistochemistry was performed on prostate tissues from 28 BPH patients and mice to assess CXCL13, p16, and CD4 expression. After deparaffinization and graded ethanol rehydration, antigen retrieval was conducted in citrate buffer (pH 6.0) for 20 min. Endogenous peroxidase was blocked with 3% H_2_O_2_, and non‐specific binding was reduced with 5% BSA.

Sections were incubated overnight at 4°C with primary antibodies: anti‐CXCL13 (1:100, ab317508), anti‐p16 (1:50, ab51243), and anti‐CD4 (human: 1:500, ab133616; mouse: 1:50, sc‐19641). After PBS washes, biotin‐conjugated secondary antibodies and an ABC kit were used for amplification. DAB served as the chromogen, and sections were counterstained with hematoxylin.

### 2.12 Scoring of IHC Staining

IHC staining was quantified using optical density (OD) and a semi‐quantitative 0–3 scoring system. For human samples, three fields per section were analyzed; for mouse tissues, five. Images were captured via brightfield microscopy and analyzed with Image‐Pro Plus v6.0. OD values were reported as 1 × 10^5^ units. Two blinded pathologists (Z.Liu. and X.W.) independently scored staining intensity. Samples were grouped by median p16 OD into high‐ and low‐senescence cohorts. CXCL13 and CD4 expression were compared using violin plots, with group differences evaluated via Student's *t*‐test or one‐way ANOVA.

### 2.13 Cell Culture and Treatment

BPH‐1 cells (Cat# KG1008, KeyGEN), an immortalized human BPH epithelial line, and primary PrECs isolated from TURP‐derived prostate tissue were used. Tissues were minced and digested with 10 mg/mL collagenase I and 0.25% trypsin at 37°C (70 rpm, 2–4 h), followed by filtration and centrifugation. Cells were resuspended in DMEM/F12, and ECs were enriched using EpCAM MicroBeads (Miltenyi Biotec). After 24 h, non‐adherent cells were removed, and epithelial colonies were cultured for experiments.

Both BPH‐1 and PrECs were maintained in RPMI‐1640 with 10% FBS, 100 U/mL penicillin, and 100 μg/mL streptomycin at 37°C in 5% CO_2_.

### 2.14 Senescent Epithelial Cell Models

Senescence was induced by treating BPH‐1 and PrECs with 25 μM cisplatin for 24 h after 6 h and 12 h of adherence, respectively. Cells were then cultured in fresh medium for 24, 48, or 96 h before analysis (Saleh et al. 2020; Sriratanasak et al. 2022; Zhao et al. 2004). After fixation with 4% paraformaldehyde for 10 min, SA‐β‐gal staining was performed overnight at 37°C. Senescent cells (blue/green) were counted under a brightfield microscope, and senescence was quantified as the percentage of SA‐β‐gal^+^ cells.

### 2.15 Cell Viability Assay

Senescence was induced in BPH‐1 and PrECs with 25 μM cisplatin for 24 h following 6 h and 12 h of adherence, respectively. Cells were then cultured in fresh medium for 24, 48, or 96 h. Proliferation was assessed using the CCK‐8 assay (Cat#KGA317‐2, KeyGEN). Cells were seeded at 3000 per well in 96‐well plates, treated as described, and incubated with 10 μL CCK‐8 reagent for 2 h at 37°C. Absorbance at 450 nm was measured, and viability was calculated relative to untreated controls.

### 2.16 DNA Synthesis Analysis

DNA synthesis was assessed using the EdU incorporation assay (Cell‐Light EdU Apollo567 Kit, RIBOBIO). Cisplatin‐treated BPH‐1 and PrECs were incubated with 10 μM EdU for 2 h, fixed with 4% paraformaldehyde, and permeabilized with 0.5% Triton X‐100. EdU incorporation was detected via Click‐iT chemistry per the manufacturer's instructions. Nuclei were counterstained with DAPI, and EdU^+^ cells were quantified using a Leica TCS SP8 MP FLIM system.

### 2.17 Enzyme‐Linked Immunosorbent Assay (ELISA)

Cytokine levels in culture supernatants were measured using Quantikine ELISA kits (R&D Systems) for human IL‐1α (DLA50), IL‐6 (D6050), TNF‐α (DTA00D), and CXCL13 (DCX130). Supernatants were collected post‐treatment, centrifuged at 1000 × g for 10 min, and stored at −80°C. Absorbance at 450 nm was measured, and cytokine concentrations were calculated from standard curves using kit‐provided recombinant standards.

### 2.18 Western Blot Analysis

Total protein was extracted from cisplatin‐treated and control cells using RIPA buffer with protease inhibitors (Roche). Protein concentrations were measured by BCA assay (Thermo Fisher). Equal amounts (30 μg) were separated by SDS‐PAGE and transferred to PVDF membranes (Millipore).

Membranes were blocked with 5% skim milk in TBS‐T and incubated overnight at 4°C with primary antibodies against p16, Rb, CDK4, CDK6, Cyclin D1, and β‐tubulin (loading control; antibody details in Supplementary Detailed Methods). After washing, HRP‐conjugated secondary antibodies were applied for 1 h at room temperature. Bands were visualized using ECL (Thermo Fisher).

Densitometry was performed with ImageJ, and protein levels were normalized to β‐tubulin. Experiments were repeated in triplicate, and results are presented as mean ± SD.

### 2.19 Quantitative Real‐Time PCR (qRT‐PCR)

Total RNA was extracted from BPH‐1 and PrEC cells using TRIzol (Invitrogen) and quantified with a NanoDrop (Thermo Fisher). cDNA was synthesized using EasyScript SuperMix (TransGen Biotech), and qRT‐PCR was performed on an ABI 7500 system using TransStart Top Green qPCR SuperMix with a two‐step protocol. Expression of p16, Rb, IL‐1α, and IL‐6 was normalized to GAPDH using the 2^−ΔΔCt method.

Primer Sequences: GAPDH: F 5′‐ACAACTTTGGTATCGTGGAAGG‐3′, R 5′‐GCCATCACGCCACAGTTTC‐3′

p16: F 5′‐ATGGAGCCTTCGGCTGACT‐3′, R 5′‐GTAACTATTCGGTGCGTTGGG‐3′

Rb: F 5′‐GCGGAGTCCAAATTCCAACAG‐3′, R 5′‐TGTCCCGAGGGTCTACAGTG‐3′

IL‐1α: F 5′‐CCCAGATCAGCACCTTACACCTA‐3′, R 5′‐CTTCTGCCTGACGAGCTTCAT‐3′

IL‐6: F 5′‐ATGTAGCCGCCCCACACA‐3′, R 5′‐CCAGTGCCTCTTTGCTGCTT‐3′

### 2.20 Flow Cytometric Analysis of Immune Cell Subsets in Human and Mouse BPH Tissue

#### 2.20.1 Tissue Processing and Immune Cell Isolation

Fresh BPH tissues from TURP procedures were processed within 2 h. After removing connective and necrotic tissue, samples were minced into 2–4 mm fragments and digested in RPMI‐1640 containing 10 mg/mL collagenase I and 0.25% trypsin at 37°C with gentle shaking (70 rpm) for 2–4 h. The digested suspension was filtered through a 70 μm strainer, washed, and resuspended in FACS buffer (PBS + 2 mM EDTA + 2% FBS) for flow cytometry.

#### 2.20.2 Surface Marker Staining and Immune Subset Identification

Dead cells were excluded using the Zombie Violet Fixable Viability Kit (BioLegend, Cat# 423113). Human immune cells were stained with the following fluorochrome‐conjugated monoclonal antibodies (all from BioLegend): CD45‐PerCP‐Cy5.5, clone HI30 (Cat# 368503); CD3‐APC, clone UCHT1 (Cat# 300411); CD4‐APC‐Cy7, clone OKT4 (Cat# 300517); CD8a‐FITC, clone RPA‐T8 (Cat# 300905); CD19‐PE, clone HIB19 (Cat# 302208). The gating strategy included lymphocytes (FSC/SSC), singlets, live cells (Zombie Violet‐negative), and CD45^+^ cells. Subsets were defined as CD4^+^ T cells (CD45^+^CD3^+^CD4^+^), CD8^+^ T cells (CD45^+^CD3^+^CD8^+^), and B cells (CD45^+^CD3^−^CD19^+^). Proportions were calculated as percentages of CD45^+^ cells.

#### 2.20.3 Functional Subtyping of CD4 ^+^ T Cells

Single‐cell suspensions were stimulated with PMA (50 ng/mL) and ionomycin (1 μg/mL) in the presence of Brefeldin A (10 μg/mL, BioLegend, Cat# 420601) for 4–6 h at 37°C and 5% CO_2_. Cells were fixed and permeabilized using the Intracellular Fixation & Permeabilization Buffer Set (BioLegend, Cat# 421002), then stained with intracellular antibodies (all from BioLegend): Perforin‐APC, clone B‐D48 (Cat# 308107); FOXP3‐APC, clone 206D (Cat# 320113); IFN‐γ‐FITC, clone 4S.B3 (Cat# 506505); IL‐4‐FITC, clone MP4‐25D2 (Cat# 500806); and IL‐17A‐FITC, clone BL168 (Cat# 512305). Data were acquired using a BD LSRFortessa flow cytometer and analyzed in FlowJo v10.8.1. CD4^+^ T cell subsets were gated within CD3^+^CD4^+^ live cells based on marker expression.

#### 2.20.4 Mouse Immune Cell Profiling

Mouse prostate tissues were mechanically and enzymatically dissociated into single‐cell suspensions. Cells were stained with fluorochrome‐conjugated antibodies (BioLegend): CD3‐APC (Cat# 100235), CD4‐APC‐Cy7 (Cat# 100414), CD8a‐FITC (Cat# 162313), CD45‐PerCP‐Cy5.5 (Cat# 157207), and Zombie Violet Fixable Viability Kit (Cat# 423113). Data acquisition and analysis followed the same protocol as for human samples.

### 2.21 Transwell Chemotaxis Assay

A transwell migration assay was used to assess the chemotactic effect of senescent ECs on CD4^+^ T cells. Conditioned media from normal and senescent BPH‐1 and PrEC cells were added to the lower chamber of 24‐well transwell plates (8 μm pores, Corning, 07‐200‐149). CD4^+^ T cells (1 × 10^4^/well) were seeded in the upper chamber and allowed to migrate for 12 h. Migrated cells in the lower chamber were quantified by microscopy and cell counting.

### 2.22 CXCL13 Expression and Chemotaxis Inhibition Assay

CXCL13 levels in conditioned media from normal and senescent ECs were measured using an ELISA kit (ab269370, Abcam). To assess chemotaxis inhibition, anti‐CXCL13 antibody (1 μg/mL, #300‐47, Peprotech) was added to the lower chamber, while CD4^+^ T cells were pretreated with anti‐CXCR5 antibody (1 μg/mL, #V3S‐0622‐YC4407, Creative Biolabs) in the upper chamber. This setup was used to evaluate the role of CXCL13 in CD4^+^ T cell migration toward senescent epithelial cells.

### 2.23 RNA Sequencing (RNA‐Seq) Data Processing and Analysis of CD4 ^+^ T Cell Co‐Culture

Total RNA was extracted from senescent and normal PrECs co‐cultured with CD4^+^ T cells at a 1:50 ratio. mRNA libraries were prepared using oligo(dT) enrichment, fragmentation, reverse transcription, adaptor ligation, and PCR amplification and sequenced on an Illumina platform (150 bp paired‐end reads).

Raw reads were processed with Cutadapt (v1.9.1), aligned to GRCh38 using HISAT2 (v2.2.1), and quantified with HTSeq (v0.6.1). Differentially expressed genes (DEGs) were identified using DESeq2 (FDR < 0.05, |log_2_FC| > 1). GSEA was performed using clusterProfiler with gene sets from MSigDB (via msigdbr), and enriched pathways were visualized with enrichplot.

### 2.24 Flow Cytometric Cell Sorting and Cytotoxicity Assay

Human PBMCs were isolated from healthy donor blood using Ficoll‐Paque density gradient centrifugation (Biochrom, Germany). Cells were stained with Zombie Violet viability dye (Cat# 423113, BioLegend) and processed for flow cytometry to analyze CD4^+^ T cell subsets, CD8^+^ T cells, and B cells.

Experimental groups included:

Untreated control (0:1, PrECs only)

B cells (CD3^−^CD19^+^)

Unmodified CD4^+^ T cells

CD4^+^ T cells with cytotoxicity blocked by Concanamycin A (100 nM, Cat# C9705, Sigma; 1 h pre‐incubation at 37°C)

CD4^+^ T cells with Treg function blocked by anti‐CD25 (clone bc96, 5 μg/mL, Cat# 302603, BioLegend; 30 min pre‐incubation)

All immune cells were used at passages 2–6. Apoptosis was assessed using Annexin V‐FITC/PI staining (KeyGEN BioTECH) and analyzed on a FACS Calibur (BD) with FlowJo v10.8.1.

To assess HLA‐DR involvement in immune‐mediated apoptosis, ECs (normal or senescent) were pretreated with anti‐HLA‐DR antibody (100 μg/mL, Cat# BE0306, Bio X Cell) or isotype control for 12 h. Co‐culture with autologous CD4^+^ T cells was then performed at E:T ratios of 0:1, 10:1, 50:1 + isotype, and 50:1 + anti‐HLA‐DR, followed by Annexin V/PI staining.

### 2.25 Animal Experiment Design

Eight‐week‐old male BALB/c mice were housed under SPF conditions with free access to food and water. After a one‐week acclimation, the mice were anesthetized with pentobarbital sodium and underwent bilateral orchiectomy. Following a one‐week recovery, the mice were randomly assigned to four groups (*n* = 8 each): control, BPH, BPH + rmCXCL13, and BPH + rmCXCL13 + anti‐CD4.

Except for the control group, all mice received daily subcutaneous injections of testosterone propionate (5 mg/kg; T7260, Solarbio) to induce BPH. Recombinant murine CXCL13 (1 μg/day; HY‐P77911, MedChemExpress) was administered intraperitoneally on days 1, 5, 8, 12, 15, and 19. CD4^+^ T cell depletion was achieved in the BPH + rmCXCL13 + anti‐CD4 group using anti‐CD4 antibody (200 μg/day; BE0003‐1, Bio X Cell) on the same days.

After three weeks, mice were euthanized, and prostate tissues were collected for analysis. All procedures were approved by the Animal Care and Use Committee of Peking University First Hospital.

### 2.26 Prostate Index Measurement

Mice were euthanized, and prostates were excised, weighed, and used to calculate the prostate index (prostate weight/body weight) as a measure of BPH severity.

### 2.27 Statistical Analysis

Data are presented as mean ± SD from at least three independent experiments. Group comparisons were performed using one‐way ANOVA with Tukey's post hoc test. Pearson's correlation was used to assess relationships among CXCL13 OD, p16 expression, and CD4^+^ T cell infiltration.

For scRNA‐seq data, DEG analysis was conducted using pseudobulk aggregation in Seurat followed by the limma‐voom pipeline. For datasets lacking biological replicates (e.g., mouse *n* = 1/group or paired senescent vs. non‐senescent regions), limma‐voom or Seurat's FindMarkers() (Wilcoxon test) was applied with caution. DEGs were filtered at FDR‐adjusted *p* < 0.05.

All Analyses Were Performed Using GraphPad Prism v9.0 or R v4.3.0, with *p* < 0.05 Considered Significant

## 3 Results

### 3.1 scRNA‐Seq Reveals That Senescent ECs in BPH Actively Recruit CD4+ T Cells via CXCL Signaling

scRNA‐seq of prostate tissues from six BPH patients (GSE226237) identified 27,146 cells across 20 populations, including epithelial (2669), CD8^+^ T (10,700), CD4^+^ T (2863), myeloid (4454), fibroblasts (517), mast (487), NK (1889), endothelial (1180), myofibroblasts (475), and B cells (1912) (Figure S1a–c). UMAP projections revealed functional heterogeneity within these cell types (Figure S1d,e). Cell type annotation was based on canonical marker genes, which are listed in Table S2.

**FIGURE 1:**
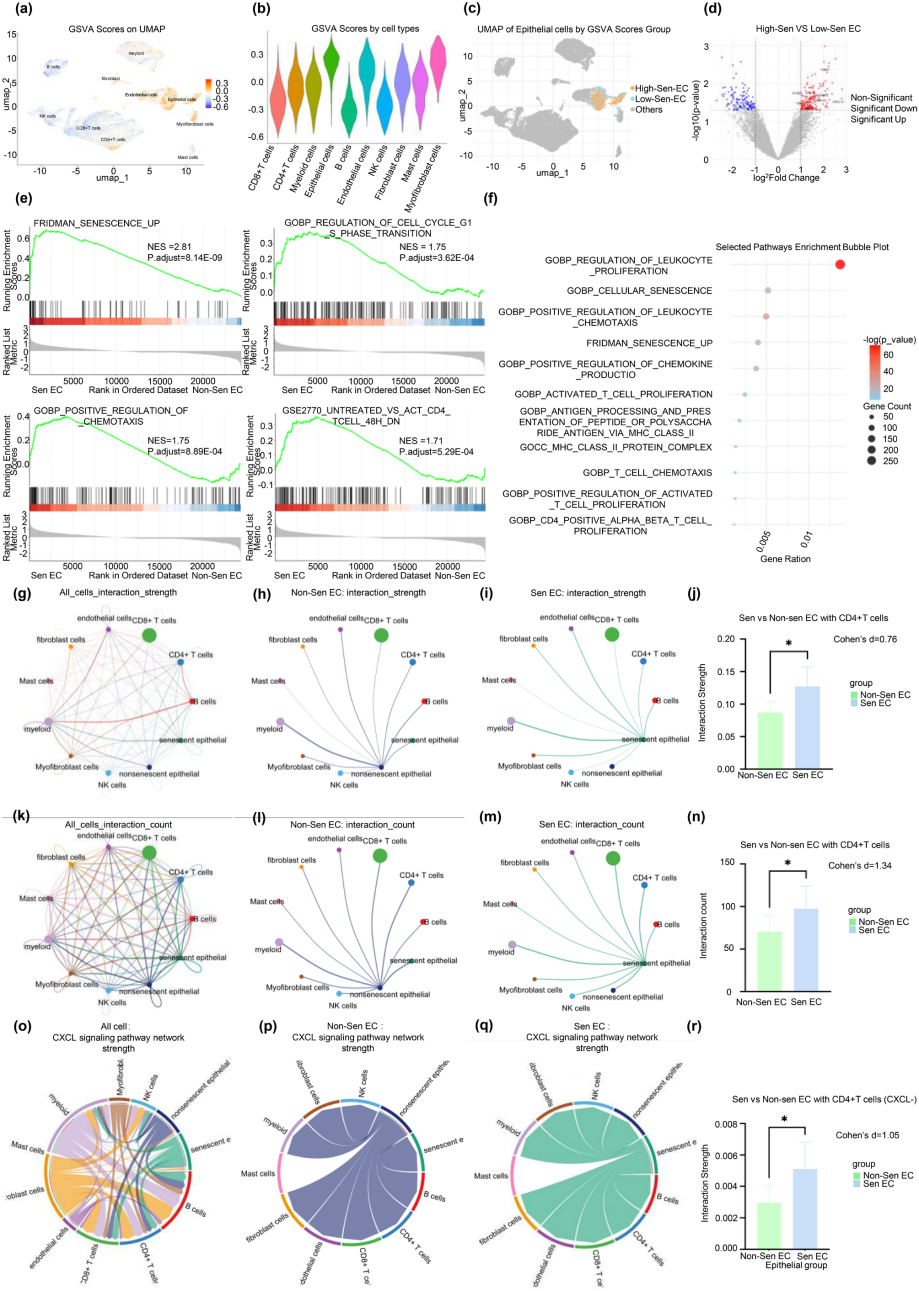
GSVA and Cellchat analysis uncover senescence‐driven CXCL signaling and CD4+ T Cell recruitment in BPH senescent epithelial cells. (a) UMAP plot illustrating the GSVA scores for the FRIDMAN_SENESCENCE_UP gene set across all cell types in the BPH microenvironment. Epithelial cells, endothelial cells, and myofibroblasts exhibit higher senescence scores, indicating a greater susceptibility to senescence compared to other cell types. (b) Violin plots showing the distribution of FRIDMAN_SENESCENCE_UP GSVA scores across the major cell types, highlighting the significantly elevated senescence scores in epithelial, endothelial, and myofibroblast cells. (c) UMAP plot displaying the spatial distribution of epithelial cells stratified into high‐ and low‐senescence groups. The distinct separation of these groups suggests that senescence status significantly influences the functional diversity of epithelial cells. (d) Volcano plot depicting differentially expressed genes (DEGs) between high‐Sen and low‐Sen epithelial groups. Several senescence‐associated genes—IGFBP2, STAT1, SPART, TGFB1I1, CCND1, and CCL2—are markedly upregulated in the High‐Sen group, indicating activation of SASP and cell cycle arrest mechanisms. (e) Gene set enrichment analysis (GSEA) results showing strong enrichment of the FRIDMAN_SENESCENCE_UP gene set and cell cycle‐related pathways, including GOBP_REGULATION_OF_CELL_CYCLE_G1_S_PHASE_TRANSITION. Immune‐related pathways, such as GOBP_POSITIVE_REGULATION_OF_CHEMOTAXIS and GSE2770_UNTREATED_VS_ACT_CD4_TCELL_48H_DN, suggest that senescent epithelial cells may promote CD4^+^ T cell recruitment and activation. (f) Bubble plot of selected pathways, highlighting enrichment in senescence, CD4+ T cell activity, MHC‐II antigen presentation, and chemotaxis. Pathways such as GOBP Regulation of Leukocyte Proliferation and GOBP Positive Regulation of Leukocyte Chemotaxis are notably enriched, suggesting an active role for senescent epithelial cells in immune cell recruitment. (g–n) CellChat‐based interaction network comparing High‐Sen and Low‐Sen epithelial cells. Senescent epithelial cells show significantly higher interaction strength (*p* = 0.049, Cohen's *d* = 0.76) and interaction count (*p* = 0.021, Cohen's *d* = 1.34) with CD4^+^ T cell. (o–r) Further exploration of the CXCL signaling pathway reveals upregulation of CXCL family members in senescent epithelial cells, facilitating their interactions with CD4+ T cells. This demonstrates the critical role of CXCL signaling in regulating immune responses in BPH. *p < 0.05.

GSVA using the *FRIDMAN_SENESCENCE_UP* gene set revealed elevated senescence scores in epithelial, endothelial, and myofibroblast cells (Figure 1a,b), suggesting structural cell susceptibility to chronic stress‐induced senescence. ECs were stratified into High‐ and Low‐Sen groups, with UMAP projections showing distinct functional states (Figure 1c).

DEG analysis between high‐ and low‐Sen groups (Table S3) showed upregulation of key senescence/SASP genes, including *IGFBP2* (*log2 fold change* = *1.61, p* = *2.10E‐02*), *STAT1* (*log2 fold change* = *1.55, p* = *2.11E‐02*), *SPART* (*log2 fold change* = *1.29, p* = *9.50E‐03*), *TGFB1I1* (*log2 fold change* = *1.65, p* = *2.32E‐02*), *CCND1* (*log2 fold change* = *1.10, p* = *2.12E‐02*), *and CCL2* (*log2 fold change* = *2.01, p* = *2.08E‐02*) (Figure 1d, Table S3.1). GSEA confirmed enrichment of the FRIDMAN_SENESCENCE_UP gene set (NES = 2.81, *p* = 8.14E‐09), alongside cell cycle‐related pathways such as GOBP_REGULATION_OF_CELL_CYCLE_G1_S_PHASE_TRANSITION (NES = 1.75, *p* = 3.62E‐04) and immune‐related pathways including GOBP_POSITIVE_REGULATION_OF_CHEMOTAXIS (NES = 1.75, *p* = 8.89E‐04) and GSE2770_UNTREATED_VS_ACT_CD4_TCELL_48H_DN (NES = 1.71, *p* = 5.29E‐04), suggesting senescent ECs may promote CD4^+^ T cell recruitment and activation (Figure 1e, Table S4).

Bubble plots illustrated enrichment of pathways linked to senescence, MHC‐II antigen presentation, and leukocyte chemotaxis (Figure 1f, Table S5), indicating that senescent ECs may actively recruit and stimulate immune cells, particularly CD4^+^ T cells.

CellChat analysis showed increased interaction strength (*p* = 0.049, Cohen's *d* = 0.76) (Figure 1g–j) and count (*p* = 0.021, *d* = 1.34) (Figure 1k–n) between senescent ECs and CD4^+^ T cells. Further exploration of the CXCL signaling pathway revealed elevated expression of CXCL genes (*p* = 0.048, *d* = 1.064), enhancing epithelial–CD4^+^ T cell communication (Figure 1o–r).

Collectively, these results suggest that senescent ECs in BPH promote CD4^+^ T cell recruitment and activation via CXCL signaling, contributing to immune modulation in the BPH microenvironment.

### 3.2 CXCL13 Upregulation and CD4+ T Cell Pathway Enrichment in Senescent BPH Prostate ECs and Aging Prostate Tissues

**FIGURE 2:**
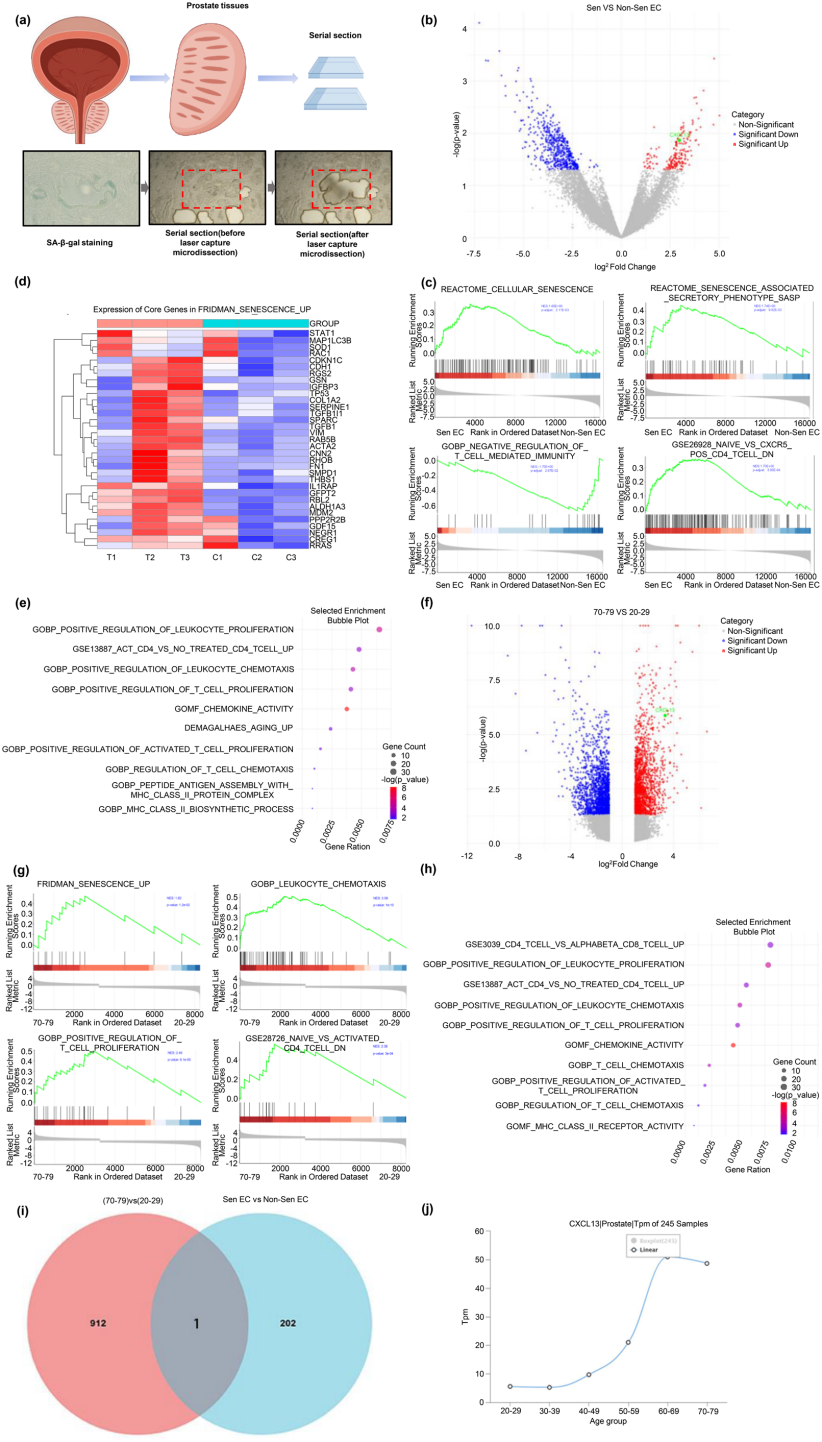
Laser capture microdissection was used to isolate senescent and non‐senescent epithelial cells in BPH for transcriptomic and pathway analysis. (a) Schematic workflow showing the isolation of paired senescent and non‐senescent epithelial cells from BPH tissue using serial sectioning, SA‐β‐gal staining, and laser capture microdissection (LCM) for downstream transcriptomic analysis. (b) Volcano plot of differentially expressed genes (DEGs) between senescent and non‐senescent epithelial cells. CXCL13 is significantly upregulated in senescent cells. (c) Gene set enrichment analysis (GSEA) reveals significant enrichment of the REACTOME_CELLULAR_SENESCENCE and REACTOME_SASP pathways in senescent epithelial cells, along with immune‐related pathways including GSE26928_NAIVE_VS_CXCR5_POS_CD4_TCELL_DN and GOBP_NEGATIVE_REGULATION_OF_T_CELL_MEDIATED_IMMUNITY, suggesting immune activation and chemotactic signaling. (d) Heatmap displaying upregulation of key senescence‐ and inflammation‐associated genes such as TP53, CDKN1C (p57), SERPINE1, and other SASP factors in senescent epithelial cells. (e) MSigDB GO enrichment analysis showing overrepresentation of pathways related to senescence, lymphocyte activation, CD4^+^ T cell proliferation, and leukocyte chemotaxis. (f) Volcano plot showing transcriptomic differences between prostate tissues from individuals aged 70–79 and 20–29. CXCL13 is notably upregulated in the elderly group. (g) GSEA plots showing enrichment of FRIDMAN_SENESCENCE_UP, GOBP_LEUKOCYTE_CHEMOTAXIS, GOBP_POSITIVE_REGULATION_OF_T_CELL_PROLIFERATION, and GSE28726_NAIVE_VS_ACTIVATED_CD4_TCELL_DN in the older cohort, indicating enhanced senescence and T cell activation in aged prostate tissue. (h) MSigDB enrichment analysis further confirms significant immune activation in aged prostates, highlighting pathways involving CD4^+^ T cell recruitment and chemokine activity. (i) Venn diagram identifying CXCL13 as the only gene consistently upregulated in both senescent epithelial cells and aged prostates (70–79 vs. 20–29), emphasizing its conserved role in senescence and immune crosstalk. (j) Independent analysis using the ADEIP dataset confirms age‐associated upregulation of CXCL13, supporting its pivotal involvement in prostate aging and BPH development.

To validate senescent ECs identified via scRNA‐seq, paired senescent and non‐senescent regions were collected from prostate tissues of three BPH patients using serial sectioning, SA‐β‐gal staining, and LCM (Figure 2a).

Transcriptome profiling revealed substantial gene expression differences, with CXCL13 (log_2_FC = 2.98, *p* = 1.36E‐02) significantly upregulated in senescent ECs (Figure 2b, Table S6). A heatmap showed elevated expression of core genes from FRIDMAN_SENESCENCE_UP, including TP53, CDKN1A, SERPINE1 (downregulated), and SASPs (Figure 2c, Table S6.1), confirming a senescent and pro‐inflammatory phenotype.

GSEA demonstrated enrichment of senescence pathways (REACTOME_CELLULAR_SENESCENCE, NES = 1.65, *p* = 0.027; REACTOME_SASP, NES = 1.74, *p* = 0.0099), indicating activation of secretory and chemotactic programs.

Notably, the immune‐related gene set GSE26928_NAIVE_VS_CXCR5_POS_CD4_TCELL_DN was enriched (NES = 1.53, *p* = 0.003), suggesting increased activation of CXCR5^+^ CD4^+^ T cells, likely driven by CXCL13 (Figure 2d).

In contrast, non‐senescent ECs were enriched in GOBP_NEGATIVE_REGULATION_OF_T_CELL_MEDIATED_IMMUNITY (NES = −1.70, *p* = 0.027), implying an immunosuppressive profile (Table S7).

Additional MSigDB enrichment analysis highlighted pathways related to lymphocyte activation, CD4^+^ T cell proliferation, and chemotaxis (Figure 2e, Table S8), reinforcing the link between senescence and CD4^+^ T cell recruitment.

Analysis of ADEIP transcriptomic data revealed significantly elevated CXCL13 (log_2_FC = 3.34, *p* = 0.000001347) expression in prostate tissues from individuals aged 70–79 compared to 20–29 (Figure 2f, Table S9), with expression increasing progressively with age (Figure 2j), implicating CXCL13 in prostate aging and BPH pathogenesis.

GSEA in the older cohort (70–79) showed enrichment in senescence‐ and immune‐related pathways, including FRIDMAN_SENESCENCE_UP (NES = 1.82, *p* = 1.2E‐02), GOBP_LEUKOCYTE_CHEMOTAXIS (NES = 3.59, *p* = 1.0E‐10), GOBP_POSITIVE_REGULATION_OF_T_CELL_PROLIFERATION (NES = 2.49, *p* = 8.1E‐05), and GSE28726_NAIVE_VS_ACTIVATED_CD4_TCELL_DN (NES = 2.38, *p* = 3.0E‐04) (Figure 2g, Table S10).

MSigDB pathway enrichment further confirmed activation of CD4^+^ T cell‐related programs—proliferation, chemotaxis, and chemokine signaling—suggesting enhanced immune cell recruitment in aged prostate tissues (Figure 2h, Table S11).

A Venn diagram comparing upregulated genes in senescent ECs (203 genes) and aged tissues (913 genes) identified CXCL13 as the sole overlapping gene (Figure 2i), emphasizing its central role in both senescence and aging‐associated immune modulation in BPH.

### 3.3 Immunohistochemical Analysis of CXCL13, p16, and CD4 in BPH Tissues Reveals an Age‐Dependent Association Between Senescent Cells and Immune Infiltration

**FIGURE 3:**
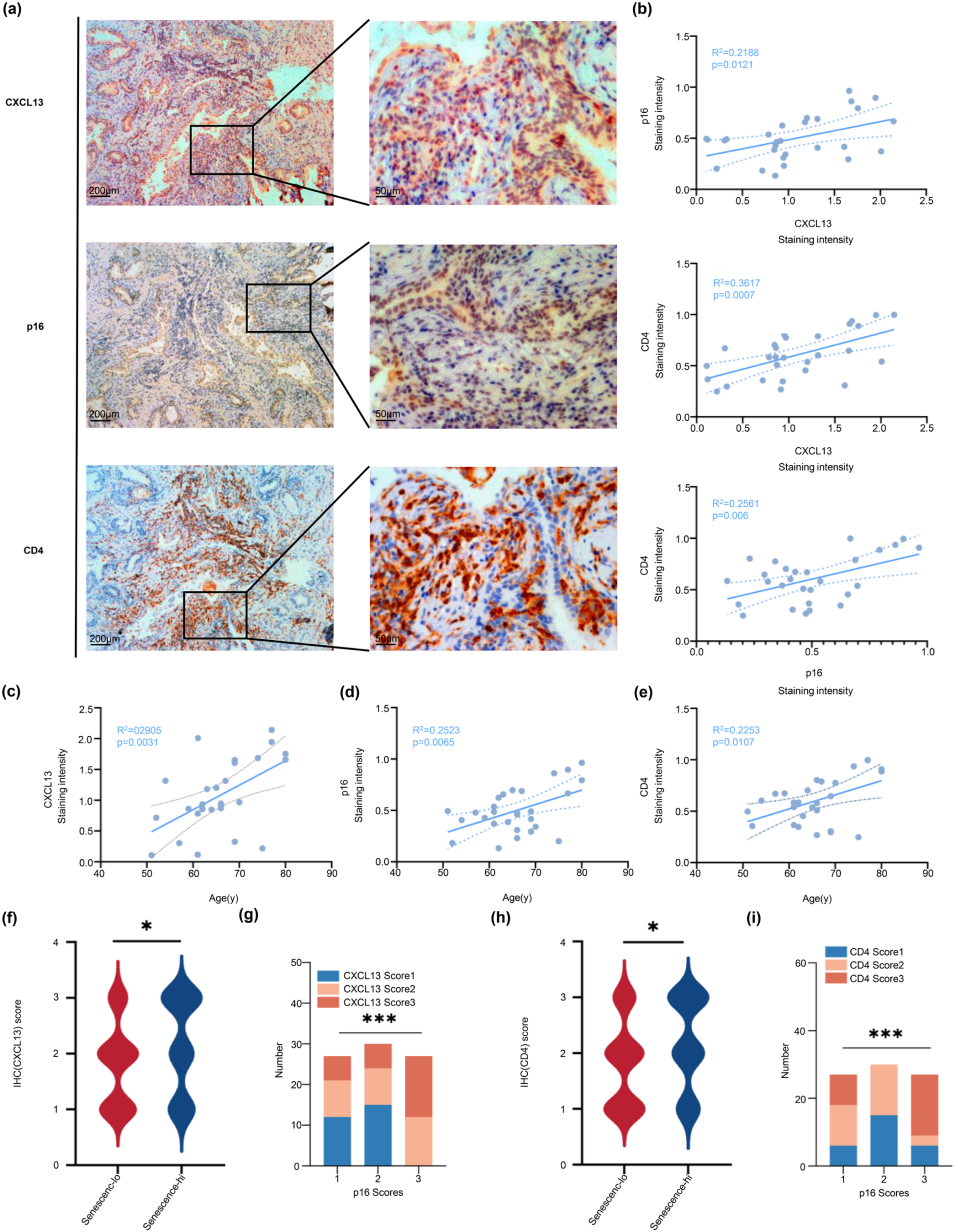
Immunohistochemical analysis of CXCL13, p16, and CD4 in BPH tissues reveals an age‐dependent association between senescent cells and immune infiltration. (a) Representative immunohistochemical (IHC) images of BPH tissue sections stained for CXCL13, p16, and CD4 show areas of high CXCL13 expression and CD4+ T cell infiltration in regions with elevated p16, a marker of cellular senescence. Images are presented at two magnifications: low magnification (scale bar = 200 μm, left) and high magnification (scale bar = 50 μm, right) to highlight areas of interest within the same field across markers. (b) Correlation analysis of IHC staining intensities (measured as optical density, OD, in units of 1 × 10^5^) between CXCL13 and p16 (top), CD4 and CXCL13 (middle), and CD4 and p16 (bottom). Significant positive correlations were observed between CXCL13 and p16 (*R* ^2^ = 0.2188, *p* = 0.012), CD4 and CXCL13 (*R* ^2^ = 0.3617, *p* = 0.0007), and CD4 and p16 (*R* ^2^ = 0.2561, *p* = 0.006), suggesting that senescent epithelial cells may recruit CD4+ T cells via CXCL13 signaling. (c–e) Scatter plots showing age‐related increases in staining intensities for CXCL13 (C; *R* ^2^ = 0.2905, *p* = 0.0031), p16 (D; *R* ^2^ = 0.2523, *p* = 0.0065), and CD4 (E; *R* ^2^ = 0.2253, *p* = 0.0107). These results indicate an age‐dependent rise in cellular senescence and CXCL13‐mediated recruitment of CD4+ T cells to senescent sites in BPH tissue. (f–i) Violin and bar plots displaying IHC scores for CXCL13 and CD4 stratified by p16 expression levels. (f) Violin plot showing significantly higher CXCL13 scores in high‐senescence (p16‐high) versus low‐senescence (p16‐low) groups (*p* < 0.05). (g) Bar plot showing the distribution of CXCL13 staining intensities across p16 scores (1, 2, and 3), with higher p16 levels corresponding to stronger CXCL13 staining (*p* < 0.001). (h) Violin plot showing significantly higher CD4 scores in high‐senescence groups (*p* < 0.001). (i) Bar plot showing the distribution of CD4 staining intensities stratified by p16 scores, with increased CD4+ T cell infiltration correlating with higher p16 expression (*p* < 0.001). Statistical significance: *p < 0.05, ***p < 0.001.

To explore the link between senescence, CXCL13 expression, and immune response in BPH, we performed IHC staining for CXCL13, p16, and CD4 on serial prostate tissue sections. Regions with high p16 expression exhibited elevated CXCL13 and CD4^+^ T cell infiltration (Figure 3a).

Correlation analysis revealed significant associations between CXCL13 and p16 (*R* ^2^ = 0.2188, *p* = 0.012), CD4 and CXCL13 (*R* ^2^ = 0.3617, *p* = 0.0007), and CD4 and p16 (*R* ^2^ = 0.2561, *p* = 0.006) (Figure 3b), suggesting that senescent ECs may recruit CD4^+^ T cells via CXCL13 signaling.

Expression of CXCL13 (*R* ^2^ = 0.2905, *p* = 0.0031), p16 (*R* ^2^ = 0.2523, *p* = 0.0065), and CD4 (*R* ^2^ = 0.2253, *p* = 0.0107) also increased with age (Figure 3c–e), indicating age‐dependent activation of senescence and immune infiltration.

Violin plots (Figure 3f,h) and semi‐quantitative scoring (0–3 scale) showed significantly higher CXCL13 and CD4 scores in p16‐high regions, with bar charts (Figure 3g,i) confirming that CXCL13 expression and CD4^+^ T cell infiltration rise with p16 levels.

These results position CXCL13 as a potential mediator of age‐related epithelial senescence and immune activation in BPH and as a candidate target for modulating the prostate immune microenvironment.

### 3.4 Cisplatin‐Induced Senescence Model in BPH‐1 and PrEC BPH Prostate Epithelial Cells

**FIGURE 4:**
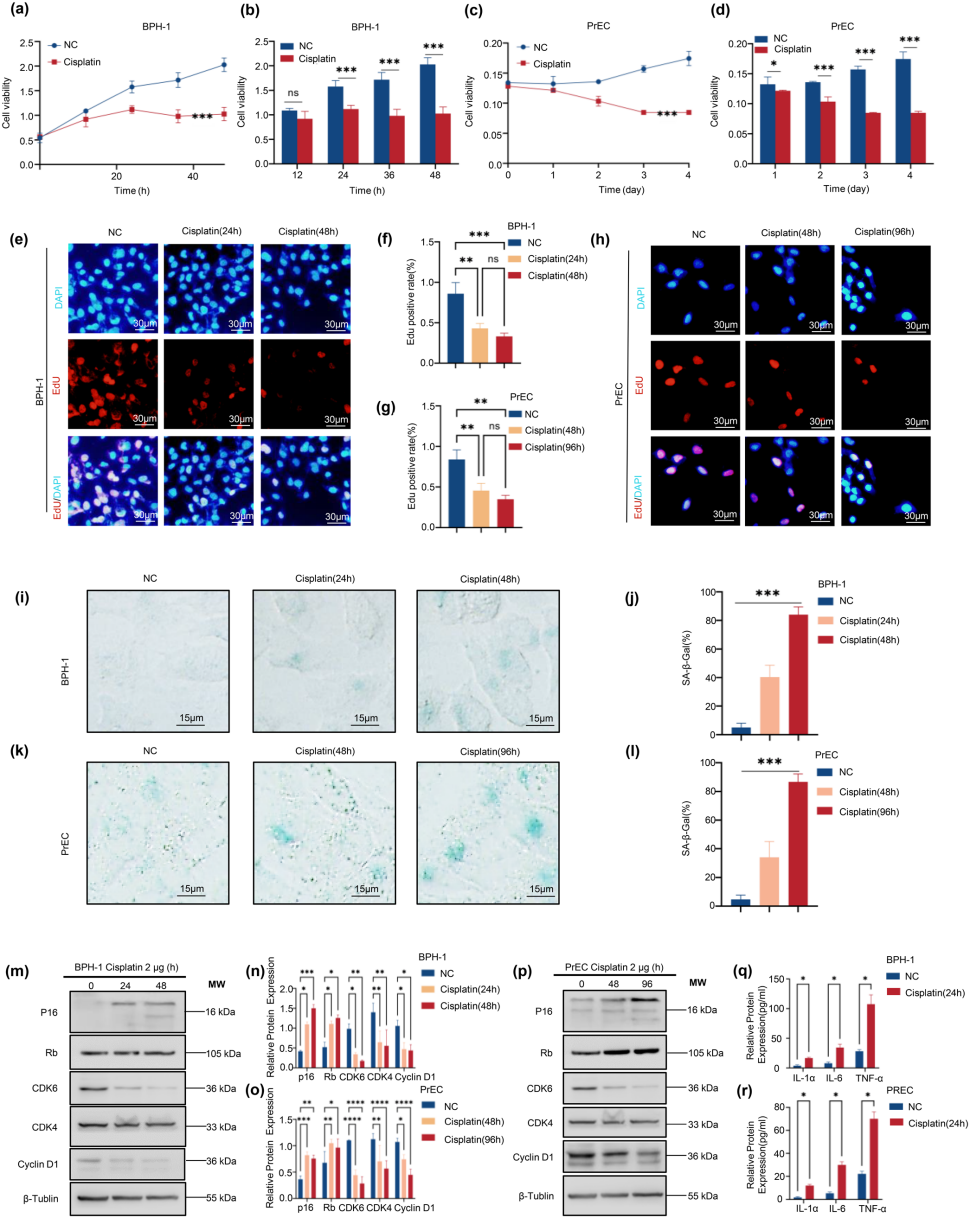
Cisplatin‐induced cellular senescence in BPH‐1 and PrEC prostate epithelial cells. (a–d) Cell viability assay (CCK‐8) in BPH‐1 (a, b) and PrEC (c, d) cells following cisplatin (25 μM/mL) treatment at various time points. Cisplatin treatment significantly inhibited cell proliferation in both cell lines. The inhibitory effects were most pronounced at 48 h in BPH‐1 cells and 96 h in PrEC cells. (e–h) EdU incorporation assay to evaluate DNA synthesis in BPH‐1 (e, f) and PrEC (g, h) cells following cisplatin treatment. A significant reduction in EdU‐positive cells was observed in both BPH‐1 and PrEC cells, indicating inhibition of DNA synthesis. (i–l) Senescence‐associated β‐galactosidase (SA‐β‐gal) staining of BPH‐1 (i, j) and PrEC (k, l) cells showing a time‐dependent increase in the percentage of SA‐β‐gal‐positive cells after cisplatin treatment, indicating the induction of cellular senescence. (m–p) Western blot analysis of cell cycle regulators p16, Rb, CDK4, CDK6, and Cyclin D1 in BPH‐1 (m, n) and PrEC (o, p) cells following cisplatin treatment. Cisplatin treatment induced an increase in p16 and Rb expression while decreasing CDK4, CDK6, and Cyclin D1 levels, suggesting cell cycle arrest. (q, r) ELISA analysis of SASP factors IL‐1α, IL‐6, and TNF‐α in BPH‐1 (q) and PrEC (r) cells following cisplatin treatment. All three cytokines were significantly elevated, consistent with a senescence‐associated secretory phenotype (SASP). Data are presented as mean ± SD; *p < 0.05. Statistical significance: **p < 0.01, ***p < 0.001, ****p < 0.0001.

For senescence induction, BPH‐1 cells were plated and allowed to adhere for 6 h before treatment with 25 μM cisplatin for 24 h. PrECs were adhered for 12 h prior to the same treatment, followed by replacement with normal growth medium and further culture for 24, 48, or 96 h before analysis. CCK‐8 assays revealed significant proliferation inhibition, peaking at 48 h in BPH‐1 and 96 h in PrEC cells (Figure 4a–d).

In BPH‐1 cells, viability declined over time: at 12 h, controls were 1.0877 ± 0.0441 vs. 0.9199 ± 0.1519 in treated cells (*p* = 0.14); at 24 h, 1.5790 ± 0.1218 vs. 1.1172 ± 0.0787 (*p* < 0.01); at 36 h, 1.7168 ± 0.1496 vs. 0.9810 ± 0.1325 (*p* < 0.01); and at 48 h, 2.0279 ± 0.1369 vs. 1.0261 ± 0.1353 (*p* < 0.001) (Figure 4a,b).

PrEC cells showed a similar trend: day 1, 0.1324 ± 0.0122 vs. 0.1217 ± 0.0010 (*p* = 0.20); day 2, 0.1360 ± 0.0012 vs. 0.1034 ± 0.0080 (*p* < 0.01); day 3, 0.1572 ± 0.0055 vs. 0.0848 ± 0.0009 (*p* < 0.0001); and day 4, 0.1744 ± 0.0119 vs. 0.0848 ± 0.0027 (*p* < 0.001) (Figure 4c,d).

EdU assays confirmed reduced DNA synthesis. In BPH‐1 cells, EdU incorporation decreased from 85.95% ± 11.17% in controls to 42.91% ± 5.16% (*p* < 0.01) at 24 h, and 33.07% ± 3.26% (*p* < 0.001) at 48 h (Figure 4e,f). In PrEC cells, incorporation dropped from 84.03% ± 9.64% to 45.50% ± 7.38% at 48 h and 34.89% ± 4.00% at 96 h (both *p* < 0.01) (Figure 4g,h).

SA‐β‐gal staining confirmed senescence: in BPH‐1 cells, positivity increased from 5.00% ± 2.45% in controls to 40.33% ± 6.80% (*p* < 0.001) at 24 h, and 84.00% ± 4.55% at 48 h (*p* < 0.001) (Figure 4i,j). In PrECs, it rose from 4.67% ± 2.49% to 34.00% ± 9.09% at 48 h and 86.67% ± 4.50% at 96 h (both *p* < 0.001) (Figure 4k,l).

Western blotting showed time‐dependent increases in p16 and Rb, alongside decreased CDK4, CDK6, and Cyclin D1, indicating G1/S arrest (Figure 4m–p). qRT‐PCR confirmed transcriptional upregulation of p16, Rb, IL‐1α, and IL‐6 in BPH‐1, 4.94 ± 1.63‐fold (*p* < 0.05), 5.23 ± 0.87‐fold (*p* < 0.01), 2.56 ± 0.60‐fold (*p* < 0.05), and 4.44 ± 1.71‐fold (*p* < 0.05), respectively; in PrECs, 7.81 ± 1.81‐fold (*p* < 0.01), 3.86 ± 0.86‐fold (*p* < 0.01), 5.79 ± 0.45‐fold (*p* < 0.001), and 3.94 ± 1.25‐fold (*p* < 0.05) (Figure 4m–p).

ELISA further confirmed SASP induction. In PrECs, cisplatin elevated IL‐1α from 1.83 ± 0.64 to 11.37 ± 1.06 pg/mL (*p* < 0.05), IL‐6 from 5.47 ± 0.84 to 30.03 ± 2.78 pg/mL (*p* < 0.05), and TNF‐α from 22.13 ± 2.35 to 70.13 ± 6.04 pg/mL (*p* < 0.05), showing ~6.2‐, 5.5‐, and 3.2‐fold increases (Figure 4q). In BPH‐1 cells, IL‐1α rose from 3.83 ± 1.54 to 16.70 ± 1.41 pg/mL (*p* < 0.05), IL‐6 from 7.93 ± 2.32 to 34.37 ± 5.79 pg/mL (*p* < 0.05), and TNF‐α from 28.23 ± 2.60 to 107.40 ± 15.95 pg/mL (*p* < 0.05), corresponding to 4.4‐, 4.3‐, and 3.8‐fold increases (Figure 4r).

Overall, cisplatin induces senescence in BPH‐1 and PrEC cells, characterized by reduced proliferation and DNA synthesis, elevated SA‐β‐gal activity, increased p16/Rb signaling, and enhanced SASP expression. This model facilitates mechanistic studies of senescence in BPH progression.

### 3.5 CXCL13–CXCR5‐Driven Recruitment and HLA‐DR‐Dependent Cytotoxicity of Senescent ECs by CD4 ^+^ CTLs Are Counteracted by Tregs

To investigate immune cell infiltration in BPH, immune cells were isolated from 13 prostate samples obtained during TURP. After enzymatic digestion and single‐cell preparation, flow cytometry was performed. Dead cells were excluded via Zombie Violet staining, and CD45^+^ leukocytes were gated for analysis. Within CD45^+^ cells, CD3^+^CD4^+^ T, CD3^+^CD8^+^ T, and CD3^−^CD19^+^ B cells were identified using fluorochrome‐conjugated antibodies.

Quantitative analysis showed CD4^+^ T cells were the predominant subset (25.74%), followed by CD8^+^ T cells (16.36%) and B cells (13.40%), suggesting a dominant role for CD4^+^ T cells in shaping the BPH immune microenvironment (Figure S5a).

**FIGURE 5:**
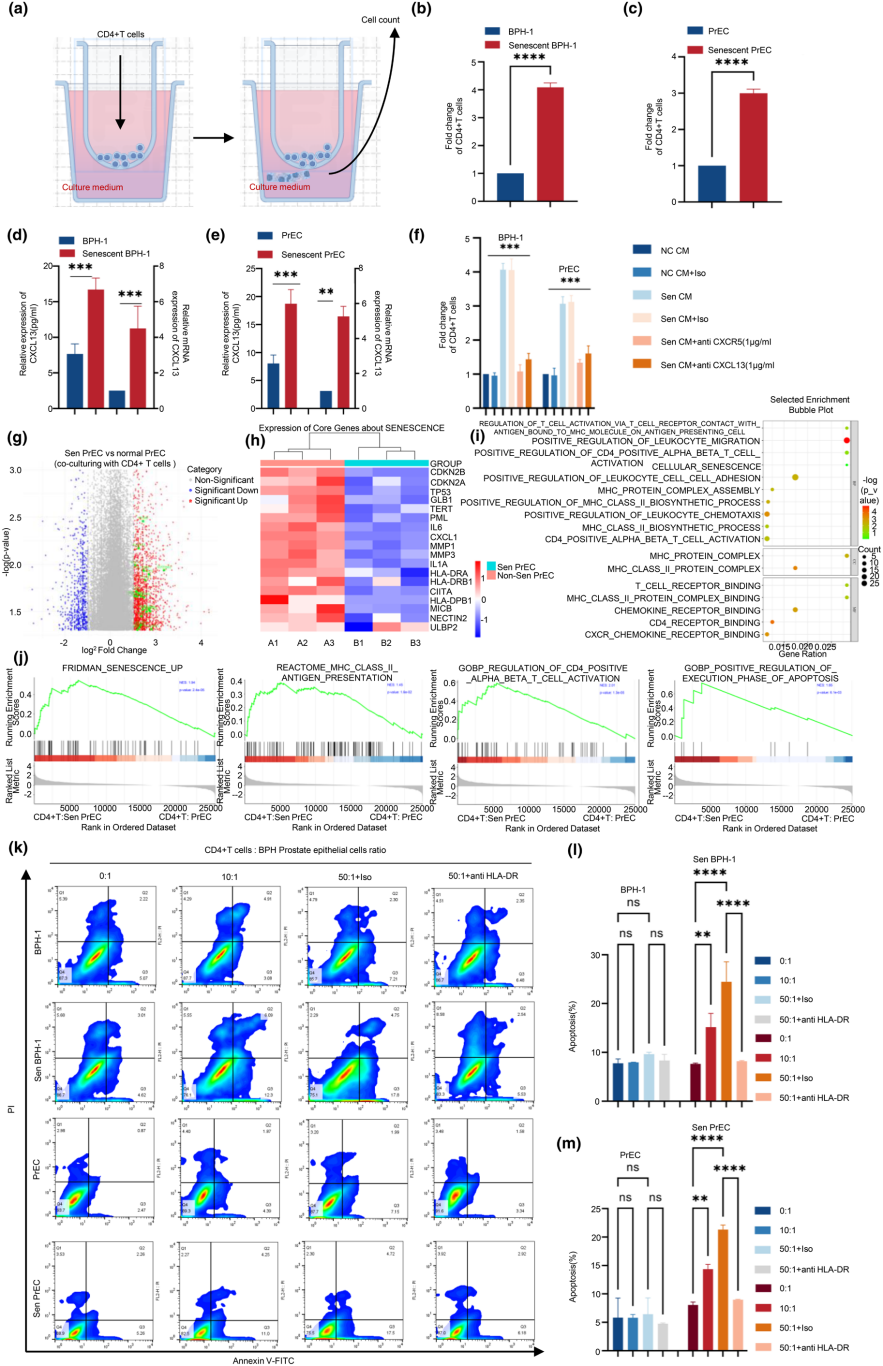
CXCL13‐CXCR5‐driven chemotaxis and HLA‐DR‐dependent apoptosis in the immune clearance of senescent BPH epithelial cells by CD4+ T cells. (a) Schematic of the transwell chemotaxis assay setup. CD4+ T cells were placed in the upper chamber, with conditioned media (CM) from senescent or normal BPH‐1 and PrEC epithelial cells in the lower chamber. CD4+ T cell migration toward senescent or normal epithelial CM was quantified after 12 h. (b, c) Fold change in CD4+ T cell migration toward conditioned media from senescent versus normal BPH‐1 (b) and PrEC (c) epithelial cells. Senescent CM significantly enhanced CD4+ T cell migration compared to normal CM. (d, e) CXCL13 expression levels in senescent and normal BPH‐1 (d) and PrEC (e) epithelial cells, as quantified by ELISA and qRT‐PCR. CXCL13 expression was significantly higher in senescent cells compared to normal cells. (f) Fold change in CD4+ T cell migration toward senescent BPH‐1 and PrEC CM, with and without anti‐CXCR5 or anti‐CXCL13 antibodies (1 μg/mL). The addition of anti‐CXCR5 or anti‐CXCL13 significantly reduced CD4+ T cell migration, demonstrating the involvement of the CXCL13‐CXCR5 axis in chemotaxis. (g) Volcano plot depicting differentially expressed genes between senescent and normal PrEC cells co‐cultured with CD4+ T cells at a 1:50 ratio. This plot highlights the upregulation of MHC‐II and SASP‐related genes, illustrating the significant changes in senescent PrEC's gene expression induced by T cell interactions. (h) Heatmap of key senescence and SASP markers in senescent versus normal epithelial cells, with significant upregulation of HLA‐DRB1, IL1A, IL1B, CXCL14, TNF, MMP13, and MMP14 in senescent cells, indicating the senescent phenotype. (i) GO enrichment analysis showing pathways related to chemokine signaling, MHC‐II antigen presentation, and T cell activation. Pathways like positive regulation of CD4+ T cell activation and T cell receptor signaling were enriched in senescent cells, supporting their role in immune interactions. (j) GSEA plots for selected pathways, including FRIDMAN_SENESCENCE_UP, REACTOME_MHC_CLASS_II_ANTIGEN_PRESENTATION, GOBP_REGULATION_OF_CD4_POSITIVE_ALPHA_BETA_T_CELL_ACTIVATION, and GOBP_POSITIVE_REGULATION_OF_EXECUTION_PHASE_OF_APOPTOSIS. These pathways are crucial in senescence, antigen presentation, and CD4+ T cell activation. (k) Flow cytometry plots showing apoptosis rates in BPH‐1 and PrEC cells (both normal and senescent) co‐cultured with CD4+ T cells at different ratios (0:1, 10:1, 50:1 + Iso, 50:1 + anti HLA‐DR). Apoptosis was measured by Annexin V‐FITC and PI staining. (l, m) Quantification of apoptosis percentages in BPH‐1 (L) and PrEC (M) epithelial cells. Apoptosis rates in senescent cells increased significantly with higher CD4+ T cell ratios. HLA‐DR inhibition at a 50:1 ratio significantly reduced apoptosis in senescent cells compared to isotype controls, highlighting the role of HLA‐DR‐mediated interactions in immune clearance. Statistical significance: *p < 0.05, **p < 0.01, ***p < 0.001, ****p < 0.0001.

To assess the chemotactic effect of senescent epithelial cells, transwell assays using conditioned media (CM) from senescent and control BPH‐1 or PrEC cells were conducted. CD4^+^ T cells exhibited enhanced migration toward senescent CM: 4.09 ± 0.13‐fold (BPH‐1) and 3.00 ± 0.09‐fold (PrEC) vs. control CM (*p* < 0.0001; Figure 5a–c).

CXCL13 was significantly elevated in senescent CM. ELISA showed protein levels increased from 7.67 ± 1.41 to 16.71 ± 1.62 pg/mL in BPH‐1 and from 8.03 ± 1.24 to 28.73 ± 2.05 pg/mL in PrEC (*p* < 0.001). qRT‐PCR confirmed transcript‐level upregulation: 4.5 ± 1.02‐fold (BPH‐1) and 5.27 ± 0.46‐fold (PrEC) (Figure 5d,e).

Blocking CXCL13 or its receptor CXCR5 markedly reduced CD4^+^ T cell migration toward senescent CM: in BPH‐1, migration dropped from 4.06 ± 0.19 to 1.08 ± 0.17 and 1.44 ± 0.14; in PrEC, from 3.06 ± 0.21 to 1.33 ± 0.08 and 1.61 ± 0.18 (all *p* < 0.001) (Figure 5f).

Bulk RNA‐seq of senescent PrEC cells co‐cultured with CD4^+^ T cells (50:1) revealed upregulation of MHC‐II genes (e.g., HLA‐DRB1, log2FC = 1.35, *p* = 3.31E‐02), senescence markers, and SASP genes: IL1B (log2 fold change = 1.81, *p* = 1.61E‐107), IL1A (log2 fold change = 1.16, *p* = 2.26E‐30), CXCL14 (log2 fold change = 3.24, *p* = 3.55E‐160), TNF (log2 fold change = 1.71, *p* = 7.62E‐04), MMP12 (log2 fold change = 2.22, *p* = 1.20E‐16), and MMP13 (log2 fold change = 2.86, *p* = 2.24E‐67) (Tables S12 and S15). GO and GSEA analyses showed enrichment in chemokine signaling, MHC‐II presentation, T cell activation, and apoptosis‐related pathways, including FRIDMAN_SENESCENCE_UP (NES = 1.94, *p* = 2.35E‐05), REACTOME_MHC_CLASS_II_ANTIGEN_PRESENTATION (NES = 1.45, *p* = 1.61E‐02), GOBP_REGULATION_OF_CD4_POSITIVE_ALPHA_BETA_T_CELL_ACTIVATION (NES = 2.01, *p* = 1.31E‐05), and GOBP_POSITIVE_REGULATION_OF_EXECUTION_PHASE_OF_APOPTOSIS (NES = 1.65, *p* = 6.13E‐03) (Figure 5g–j; Tables S13, S14).

Co‐culture (50:1) of CD4^+^ T cells with senescent ECs significantly increased apoptosis. In BPH‐1 cells, apoptosis rose from 7.64% (0:1) to 24.47% (50:1 + isotype), but declined to 8.20% upon HLA‐DR blockade. PrEC cells showed a similar pattern (Figure 5k–m).

Following PMA/ionomycin stimulation, five CD4^+^ T cell subsets were identified: cytotoxic T lymphocytes (CTLs, Perforin^+^), Tregs (FOXP3^+^), Th1 (IFN‐γ^+^), Th2 (IL‐4^+^), and Th17 (IL‐17A^+^). CD4^+^ CTLs were most abundant (15.03%), followed by Tregs (8.37%), with Th1, Th2, and Th17 comprising 4.31%, 2.11%, and 2.17%, respectively (Figure S5b).

In contrast, co‐culture with B cells had negligible effects on epithelial cell apoptosis (BPH‐1: 8.07% vs. 7.97%; PrEC: 8.09% vs. 8.07%), indicating a minimal role for B cells in senescent cell clearance.

CD4^+^ T cells substantially enhanced apoptosis in senescent ECs (20.06% in BPH‐1, 20.02% in PrEC). Inhibition of CD4^+^ CTLs with Concanamycin A reduced apoptosis to 10.25% and 10.43%, respectively—comparable to B cell conditions—highlighting CD4^+^ CTLs as the main effectors.

Conversely, blocking Treg function with anti‐CD25 increased apoptosis to 27.15% (BPH‐1) and 26.04% (PrEC), underscoring Tregs' suppressive role (Figure S5c,d).

Together, these findings reveal a functional dichotomy in CD4^+^ T cells: CTLs actively mediate senescent cell clearance via CXCL13‐CXCR5 and HLA‐DR‐dependent mechanisms, while Tregs suppress this process. B cells appear to play a limited role.

### 3.6 CXCL13 Promotes CD4+ T Cell Accumulation and Modulates Senescence Pathways in Testosterone‐Induced BPH

**FIGURE 6:**
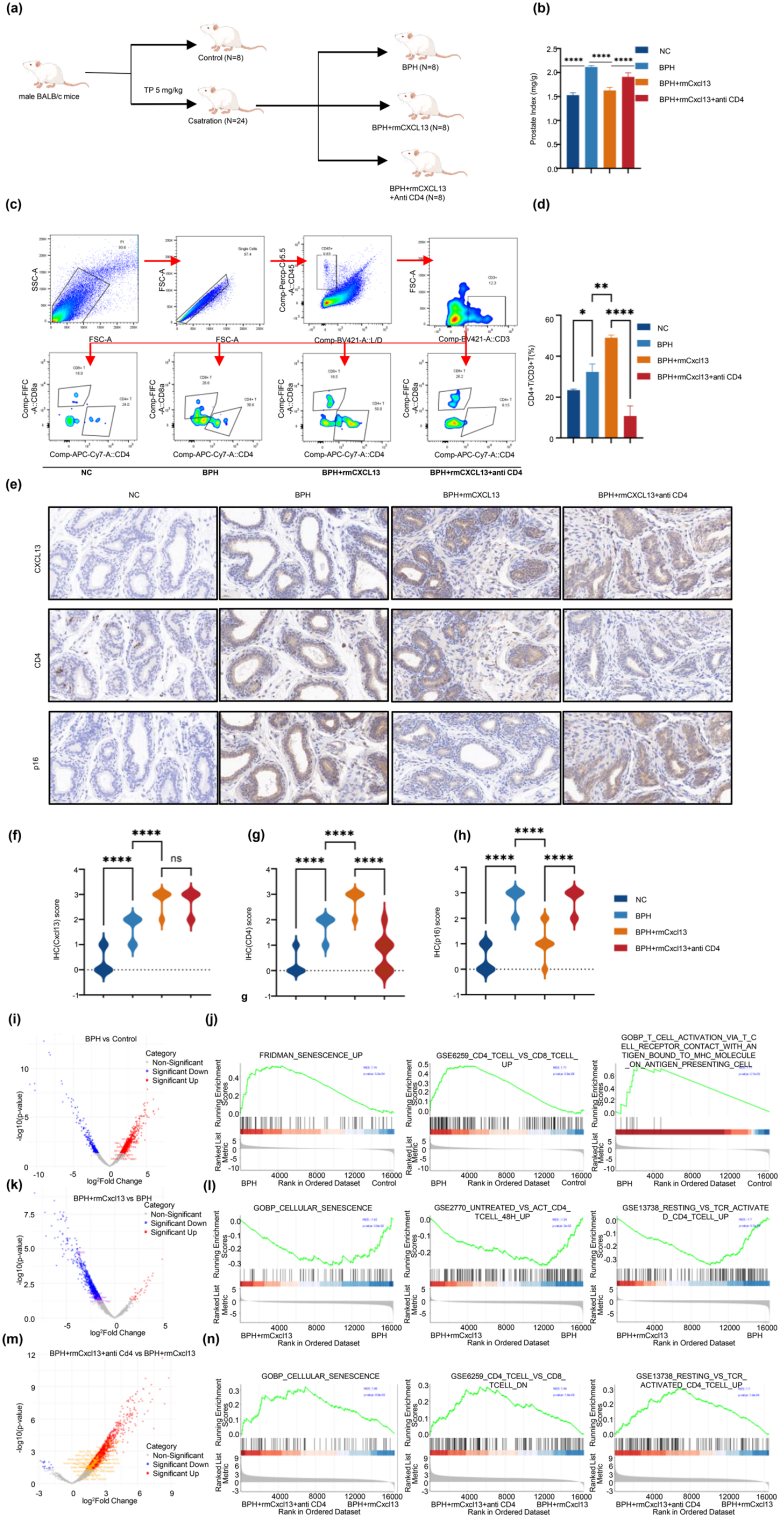
Effects of CXCL13 and CD4+ T cell depletion on prostate enlargement and senescence in a testosterone‐induced BPH model. (a) Experimental design for BPH induction in male BALB/c mice. Mice were divided into four groups: control (*n* = 8), BPH (*n* = 8), BPH + rmCXCL13 (*n* = 8), and BPH + rmCXCL13 + anti‐CD4 (*n* = 8). (b) Comparison of Prostate Index (PI) Across Groups: The PI was significantly higher in the BPH group than in the control group, confirming successful BPH induction. In the BPH + rmCXCL13 group, there was a notable reduction in PI, indicating that CXCL13 mitigates prostate enlargement. Conversely, the BPH + rmCXCL13 + anti‐CD4 group displayed a significant increase in PI relative to the BPH + rmCXCL13 group, suggesting that depletion of CD4+ T cells exacerbates CXCL13‐mitigated prostate hyperplasia. (c) Representative flow cytometry gating strategy and CD4+ T cell percentage within the CD3+ T cell population in prostate tissue across groups. (d) Quantification of CD4+/CD3+ T cell ratios across treatment groups. The BPH + rmCXCL13 group showed a significant increase in CD4+ T cell infiltration compared to the BPH group, indicating CXCL13‐mediated CD4+ T cell recruitment. In the BPH + rmCXCL13 + anti‐CD4 group, CD4+ T cells were significantly reduced, confirming effective CD4+ T cell depletion. (e) Immunohistochemical staining of prostate tissue for CXCL13, CD4, and p16 across treatment groups. CXCL13 expression increased from the control to the BPH and BPH + rmCXCL13 groups, with no significant difference between the BPH + rmCXCL13 and BPH + rmCXCL13 + anti‐CD4 groups. CD4 staining intensity was significantly higher in the BPH and BPH + rmCXCL13 groups but markedly reduced in the BPH + rmCXCL13 + anti‐CD4 group, confirming CD4+ T cell depletion. P16 staining, a marker of cellular senescence, was significantly elevated in the BPH group compared to the control. In the BPH + rmCXCL13 group, p16 expression was reduced but increased again in the BPH + rmCXCL13 + anti‐CD4 group. (f–h) Quantification of IHC scores for CXCL13, CD4, and p16 across groups, showing statistical comparisons. (i, k, m) Volcano plots of differential gene expression analyses for BPH vs. control (i), BPH + rmCXCL13 vs. BPH (k), and BPH + rmCXCL13 + anti‐CD4 vs. BPH + rmCXCL13 (m), highlighting significant changes in senescence‐related genes. (j, l, n) Gene Set Enrichment Analysis (GSEA) results for senescence and CD4+ T cell‐related gene sets. In BPH vs. control, FRIDMAN_SENESCENCE_UP, GSE6259_CD4_TCELL_VS_CD8_TCELL_UP, and GOBP_T_CELL_ACTIVATION_VIA_T_CELL_RECEPTOR_CONTACT_WITH_ANTIGEN_BOUND_TO_MHC_MOLECULE_ON_ANTIGEN_PRESENTING_CELL gene sets were significantly enriched in the BPH group. In BPH + rmCXCL13 vs. BPH, GOBP_CELLULAR_SENESCENCE, GSE2770_UNTREATED_VS_ACT_CD4_TCELL_48H_UP, and GSE13738_RESTING_VS_TCR_ACTIVATED_CD4_TCELL_UP gene sets were enriched in the BPH + rmCXCL13 group, with negative NES values indicating pathway reduction. In BPH + rmCXCL13 + anti‐CD4 vs. BPH + rmCXCL13, GOBP_CELLULAR_SENESCENCE, GSE6259_CD4_TCELL_VS_CD8_TCELL_DN, and GSE13738_RESTING_VS_TCR_ACTIVATED_CD4_TCELL_UP gene sets were enriched in the BPH + rmCXCL13 + anti‐CD4 group, indicating a potential regulatory role of CD4+ T cells in senescence signaling. *p < 0.05, **p < 0.01, ****p < 0.0001.

Male BALB/c mice were divided into four groups: control, BPH, BPH + rmCXCL13, and BPH + rmCXCL13 + anti‐CD4 (*n* = 8 per group) (Figure 6a). The prostate index (PI) was significantly elevated in the BPH group (2.12 ± 0.03) vs. controls (1.53 ± 0.05, *p* < 0.0001), confirming successful induction (Figure 6b). rmCXCL13 treatment reduced PI (1.63 ± 0.07 vs. 2.12 ± 0.03, *p* < 0.0001), suggesting suppression of hyperplasia. CD4^+^ T cell depletion reversed this effect, increasing PI to 1.91 ± 0.09 (*p* < 0.0001 vs. BPH + rmCXCL13), implicating CD4^+^ T cells in mediating CXCL13's protective effects.

Flow cytometry revealed increased CD4^+^ T cells among CD3^+^ lymphocytes across treatment groups (Figure 6c). CD4^+^/CD3^+^ ratios were 23.37% ± 0.57% (Control), 32.33% ± 3.90% (BPH), 48.97% ± 1.38% (BPH + rmCXCL13), and 10.78% ± 4.89% (BPH + rmCXCL13 + anti‐CD4) (Figure 6d). CD4^+^ infiltration was significantly higher in the rmCXCL13 group (*p* < 0.01) and reduced upon CD4^+^ T cell depletion (*p* < 0.0001), confirming both recruitment and depletion efficiency.

Immunohistochemistry for CXCL13, CD4, and p16 supported these findings (Figure 6e). CXCL13 expression increased in the BPH and BPH + rmCXCL13 groups (Figure 6f). CD4^+^ staining was elevated in the BPH and rmCXCL13‐treated groups (*p* < 0.0001) but markedly reduced in the anti‐CD4 group (*p* < 0.0001) (Figure 6g). P16 expression was upregulated in BPH (*p* < 0.0001), downregulated with rmCXCL13 (*p* < 0.0001), and re‐elevated after CD4^+^ T cell depletion (*p* < 0.0001) (Figure 6h), suggesting CD4^+^ T cells contribute to senescence suppression in the CXCL13 context.

Bulk RNA‐seq on prostate tissue from five randomly selected mice per group revealed differential expression profiles for BPH vs. control (Figure 6i, Table S16), BPH + rmCXCL13 vs. BPH (Figure 6k, Table S18), and BPH + rmCXCL13 + anti‐CD4 vs. BPH + rmCXCL13 (Figure 6m, Table S20).

GSEA of BPH vs. control identified enrichment in senescence and MHC‐II/CD4^+^ T cell‐related pathways: FRIDMAN_SENESCENCE_UP (NES = 1.74, *p* = 3.2E‐04), GSE6259_CD4_TCELL_VS_CD8_TCELL_UP (NES = 1.71, *p* = 5.8E‐06), and GOBP_T_CELL_ACTIVATION_VIA_TCR_MHC_INTERACTION (NES = 1.71, *p* = 2.7E‐03) (Figure 6j, Table S17).

In BPH + rmCXCL13 vs. BPH, senescence‐related pathways were negatively enriched: GOBP_CELLULAR_SENESCENCE (NES = –1.42, *p* = 3.6E‐02), GSE2770_UNTREATED_VS_ACT_CD4_TCELL_48H_UP (NES = –1.34, *p* = 2E‐02), and GSE13738_RESTING_VS_TCR_ACTIVATED_CD4_TCELL_UP (NES = –1.70, *p* = 5.7E‐05), indicating suppressed senescence signaling (Figure 6l, Table S19).

In BPH + rmCXCL13 + anti‐CD4 vs. BPH + rmCXCL13, these pathways were reactivated: GOBP_CELLULAR_SENESCENCE (NES = 1.58, *p* = 9.8E‐03), GSE6259_CD4_TCELL_VS_CD8_TCELL_DN (NES = 1.54, *p* = 1.6E‐03), and GSE13738_RESTING_VS_TCR_ACTIVATED_CD4_TCELL_UP (NES = 1.71, *p* = 1.9E‐04), highlighting the regulatory role of CD4^+^ T cells in suppressing senescence within the CXCL13‐induced prostate environment (Figure 6n, Table S21).

### 3.7 scRNA‐Seq Analysis and Cellular Interactions in Testosterone‐Induced BPH

scRNA‐Seq (GSM6547516, GSM6547515) from testosterone‐induced BPH and control mouse prostates revealed 11 distinct cell types via unsupervised clustering and UMAP projection (Figure S6a–c). ECs (5395), the most abundant, were marked by Epcam, Krt19, Krt8, Krt5, Krt14, and others. Fibroblasts (5337) expressed Col1a1, Col1a2, Fbln1, Lum, and Igf1. Monocytes/macrophages (2161) expressed C1qa‐c, Adgre1, Csf1r, Cd14, and Gpr34. Endothelial cells (855) expressed Vwf, Pecam1, and Cdh5. Other cell types included CD8^+^ T (403), CD4^+^ T (347), B cells (285), smooth muscle (404), myofibroblasts (175), dendritic cells (175), and mast cells (129), each identified by canonical markers (Figures S7a–d, S8a–e and S9a,b).

This cellular landscape underscores complex epithelial, stromal, and immune interactions (Figure S6d,e). GSVA using the FRIDMAN_SENESCENCE_UP gene set showed epithelial cells, fibroblasts, and monocytes/macrophages had the highest senescence scores (Figure S10a,b). ECs were further stratified into senescent and non‐senescent subsets (Figure S10c; Table S22), with UMAP projection revealing spatial localization.

Differential expression analysis identified upregulation of senescence‐associated genes in senescent epithelial cells: Cdkn1c (log2FC = 3.02, *p* = 1.61E‐107), Ccnd1 (1.91, *p* = 3.88E‐13), Igfbp3 (3.41, *p* = 3.95E‐06), Tnfaip2 (log2 fold change = 2.18, *p* = 1.54E‐05), Fn1(log2 fold change = 2.38, *p* = 1.51E‐07), Gsn (log2 fold change = 1.44, *p* = 2.18E‐12), and Cxcl14 (log2 fold change = 3.40, *p* = 2.78E‐15), indicating cell cycle arrest and matrix remodeling (Figure S10d).

GSEA revealed enrichment of FRIDMAN_SENESCENCE_UP (NES = 2.12, *p* = 1.12E‐08), GOBP_NEGATIVE_REGULATION_OF_CELL_POPULATION_PROLIFERATION (NES = 1.68, *p* = 1.12E‐08), and GOBP_POSITIVE_REGULATION_OF_CHEMOTAXIS (NES = 1.83, *p* = 9.13E‐06), suggesting senescent ECs actively suppress proliferation and recruit immune cells (Figure S10e; Table S23). Enrichment of GOBP_REGULATION_OF_EXTRINSIC_APOPTOTIC_SIGNALING_PATHWAY_VIA_DEATH_DOMAIN_RECEPTORS (NES = 1.74, *p* = 1.83E‐03) further implicates apoptosis signaling. A bubble plot highlights key enriched pathways related to senescence and CD4^+^ T cell chemotaxis (Figure S10f; Table S24).

Cell–cell communication analysis revealed stronger interactions between senescent ECs and CD4^+^ T cells compared to non‐senescent cells (Figure S10g–n). CXCL signaling analysis showed elevated interactions from senescent ECs toward CD4^+^ T cells (Figure S10o–r), suggesting active immune recruitment.

To further delineate this interaction, ECs were subclassified into luminal, basal, and neuroendocrine types (Figure S11a). GSVA showed all subtypes in BPH had elevated senescence signatures, with luminal cells showing the highest proportion (39.1%), followed by basal (32.6%) and neuroendocrine (28.3%) (Figure S11b,g).

Volcano plots comparing senescent and non‐senescent ECs in BPH revealed downregulation of Cdk5rap3 (log2FC = −0.33, *p* = 8.69E‐07) and upregulation of Cdkn1c (3.30, *p* = 3.71E‐08), along with Sparc (log2 fold change = 1.16, *p* = 1.93E‐21), Igfbp7 (log2 fold change = 2.95, *p* = 2.35E‐36), Igfbp5 (log2 fold change = 3.70, *p* = 1.32E‐31), and Thbs1(log2 fold change = 4.30, *p* = 7.49E‐21), implicating ECM remodeling and senescence signaling (Figure S11c,d; Table S25).

GSEA further confirmed enrichment of FRIDMAN_SENESCENCE_UP (NES = 1.89E+00, *p* = 5.62E‐05), GOBP_NEGATIVE_REGULATION_OF_MITOTIC_CELL_CYCLE (NES = 1.38E+00, *p* = 5.83E‐02), WU_CELL_MIGRATION (NES = 1.86E+00, *p* = 1.10E‐07), and GSE17974_0H_VS_24H_IN_VITRO_ACT_CD4_TCELL_UP (NES = 1.43E+00, *p* = 3.95E‐03) (Figure S11e; Table S26).

Finally, an enrichment bubble plot highlights upregulation of pathways related to cell cycle inhibition and immune activation, including GOBP_REGULATION_OF_MITOTIC_CELL_CYCLE, GOBP_MITOTIC_CELL_CYCLE_PHASE_TRANSITION, GOBP_T_CELL_PROLIFERATION, and GOBP_CHEMOKINE_PRODUCTION, suggesting that senescent ECs both halt their own proliferation and promote CD4^+^ T cell recruitment (Figure S11f; Table S27).

## 4 Discussion

Our study identifies a novel “immune–senescence” axis in BPH, wherein senescent ECs actively shape the immune microenvironment by secreting CXCL13. CXCL13 was markedly upregulated in aged and hyperplastic prostates and served as a key SASP factor recruiting CXCR5 +^+^ CD4 ^+^ T cells. Although traditionally associated with B cell chemotaxis (Havenar‐Daughton et al. 2016), our findings suggest B cells contribute minimally to senescent cell clearance, with CD4 ^+^ T cells playing the dominant role.

Within BPH lesions, CD4 ^+^ T cells were functionally diverse. We identified a cytotoxic subset (CD4 ^+^ CTLs) expressing perforin, likely responsible for clearing senescent ECs via MHC‐II recognition. This mirrors immune surveillance in aged skin, where CD4 ^+^ CTLs eliminate senescent fibroblasts (Hasegawa et al. 2023; Wirth et al. 2023), suggesting a similar mechanism operates in the aging prostate.

Conversely, CD4 ^+^ FoxP3 ^+^ regulatory T cells (Tregs), which accumulate with age and chronic inflammation (Han et al. 2009; Hwang et al. 2009), are also enriched in BPH. Tregs suppress cytotoxic responses and may protect senescent cells from immune clearance—mirroring mechanisms observed in aging and cancer (Barbé‐Tuana et al. 2020; Norström et al. 2016). Our study is the first to reveal this functional dichotomy of CD4 ^+^ T cells in BPH, suggesting immune evasion by senescent cells may result from active suppression rather than immune dysfunction alone.

Interestingly, despite CXCL13's classical role in B cell chemotaxis, B cells were not significantly increased in BPH, aligning with prior reports (Middleton et al. 2019). This implies CXCL13 mainly recruits CXCR5 ^+^ CD4 ^+^ T cells—such as follicular helper‐like or cytotoxic subsets—rather than B cells. A similar phenomenon occurs in aged salivary glands, where epithelial CXCL13 attracts CD4 ^+^ T cells linked to senescence (Kurosawa et al. 2021). Given B cells' limited cytotoxic function, senescent cell clearance in BPH appears largely T cell‐mediated. We propose that epithelial CXCL13 serves as a “help signal” to recruit CD4 ^+^ T cells, with clearance determined by the balance between CTLs and Tregs—not by B cell involvement.

Our study builds upon prior work linking chronic inflammation and SASP‐driven immune infiltration to BPH (Vital et al. 2014; Jiang et al. 2019), identifying CXCL13 as a previously underappreciated SASP chemokine in this context. Highly upregulated in BPH, CXCL13 mediates adaptive immune communication by recruiting CD4 ^+^ T cells to senescent epithelial regions. This is the first direct evidence of senescent prostate cells actively engaging T cells for potential clearance—highlighting a dual role for immune recruitment: clearance or suppression.

While most studies have focused on innate immunity or undifferentiated T cell responses (Li et al. 2021), our work emphasizes CD4 ^+^ T cell subsets and their crosstalk with senescent epithelia. The presence of cytotoxic CD4 ^+^ T cells (CTLs) aligns with findings from other tissues (Marin et al. 2023), and the observed accumulation of Tregs parallels immunosuppressive patterns in cancer and aging. Notably, the dynamic between CD4 ^+^ CTLs and Tregs in senescent cell clearance has not been previously described in the prostate. Our findings reveal that immune surveillance and evasion—hallmarks of cancer—also operate in benign, age‐related prostatic growth.

In summary, our findings suggest that BPH involves not only hormonal and stromal factors but also immune regulation, where the fate of senescent cells is shaped by the balance between cytotoxic CD4^+^ T cells and immunosuppressive Tregs.

Despite this advance, several limitations remain. First, our conclusions regarding the roles of CD4^+^ CTLs and Tregs are primarily based on co‐localization and functional in vitro assays. However, direct in vivo evidence—such as Treg depletion or CTL activation—is still lacking. Additionally, the contributions of NK cells and macrophages to senescent cell clearance remain underexplored (Kale et al. 2020; Moiseeva, Cisneros, Sica, et al. 2023).

Second, the mechanisms of immune evasion by senescent prostate cells require further investigation. Previous studies suggest that senescent cells may express immune checkpoint molecules like HLA‐E or PD‐L1 to avoid immune clearance (Pereira et al. 2019; Salminen 2024). The enrichment of Tregs in BPH further supports the presence of an immunosuppressive niche that may promote immune escape.

Third, mouse strain differences may impact immune phenotypes and should be considered when interpreting results. BALB/c mice, while suitable for mechanistic studies due to their defined genetic background, have low NK cell activity, potentially underestimating innate responses. In contrast, ICR mice, as an outbred strain, exhibit greater immunological heterogeneity and more closely resemble human immune variability (Shkurnikov et al. 2023; Watanabe et al. 2004). These strain‐dependent differences could influence the CXCL13–CD4^+^ T cell axis, particularly in immune infiltration and age‐associated inflammation.

Fourth, for human scRNA‐seq data, we employed a pseudobulk aggregation strategy (AggregateExpression) to ensure statistical robustness. However, due to the limited number of mouse samples (*n* = 2), this approach was not feasible, reducing the statistical power of the mouse analysis. Consequently, transcriptional trends in the mouse model should be interpreted with caution. Future studies with larger sample sizes will improve the reliability of these findings.

In summary, CXCL13 serves as a central chemokine linking senescent prostate ECs to adaptive immunity in BPH. Together with HLA‐DR–mediated antigen presentation, the CXCL13 axis helps direct the balance between cytotoxic CD4^+^ T cells and immunosuppressive Tregs, ultimately determining whether senescent cells are eliminated or persist. This insight offers a novel immunological perspective on BPH and aging‐related diseases, suggesting that modulating the CXCL13–CXCR5 axis—either by inhibiting Treg recruitment or enhancing CD4^+^ CTL function—could improve immune‐mediated clearance of senescent cells. Future strategies may include targeted immunotherapies, such as local checkpoint blockade or engineered T cells, and combinatorial approaches with senolytics or senescence‐targeted vaccines (immune senolysis) to promote tissue repair and limit chronic inflammation. Overall, this study lays the foundation for immune‐based interventions in BPH and highlights immune surveillance as a therapeutic frontier in age‐associated pathologies.

## Author Contributions

W.Y. and Q.Z. conceived and designed the study. Z.L. conducted laboratory work and data analysis and prepared the original manuscript draft. Z.L., Z.Z., X.W., S.L., C.H., Y.L., X.T., J.Z., P.Z., Y.G., Y.F., and Y.M. contributed to sample collection and data organization. W.Y. and Q.Z. provided clinical assistance and valuable insights throughout the study. They also critically advised and revised the manuscript. W.Y., Q.Z., S.L., Y.M., and K.Y. secured financial support. All authors reviewed and approved the final manuscript.

## Ethics Statement

This study was conducted in strict accordance with the principles of the Declaration of Helsinki and relevant ethical guidelines. The use of human specimens was reviewed and approved by the Ethics Committee of Peking University First Hospital. Written informed consent was obtained from all participants prior to specimen collection. All animal experiments were performed in compliance with international guidelines for the care and use of laboratory animals and were approved by the Animal Ethics Committee of Peking University First Hospital. Every effort was made to minimize the number of animals used and to reduce their suffering.

## Conflicts of Interest

The authors declare no conflicts of interest.

## Supporting information


**Figure S1.** Single‐cell RNA sequencing of prostate tissues from six BPH patients reveals distinct cell populations and functional heterogeneity. (a) UMAP plot showing the clustering of 27,146 cells from six BPH patients into 20 distinct cell populations. Each color represents a unique cluster identified based on transcriptomic similarity. (b) UMAP plot displaying the origin of cells from six individual BPH samples (L1‐S3), indicating the shared and unique cellular distributions across different patients. (c) Feature plots illustrating the expression of key marker genes across the UMAP projections. Marker genes include CD79A (B cells), LYZ (myeloid cells), DCN (fibroblasts), VWF (endothelial cells), KLK3 (epithelial cells), ACTA2 (myofibroblasts), GNLY (NK cells), CD3D (T cells), CD4 (CD4+ T cells), KIT (mast cells), and CD8B (CD8+ T cells), confirming the identity of each cell population. (d) Dot plot summarizing the expression of marker genes across the identified cell types. The size of the dots represents the percentage of cells expressing each gene within the population, and the color intensity represents the average expression level of each marker. (e) UMAP plot highlighting the distinct clustering of major cell types, including epithelial cells, CD8+ T cells, CD4+ T cells, myeloid cells, fibroblasts, mast cells, NK cells, endothelial cells, myofibroblasts, and B cells, indicating functional heterogeneity across these populations in BPH tissues.

**Figure S2.** Canonical marker gene expression across major cell types in the human BPH single‐cell dataset. Violin plots showing expression of canonical markers used to annotate the major cell lineages in the integrated scRNA‐seq dataset: (a) CD8^+^ T cells (CD3D, CD3E, CD3G, CD8A, CD8B, GZMK), (b) CD4^+^ T cells (CD4, IL2RA, ICOS, FOXP3, IL7R, CTLA4, CD40LG), (c) Myeloid cells (C1QA, C1QB, C1QC, CSF1R, MS4A6A, MS4A4A, CD14, CD68, CCR2, CD209, LYZ), (d) Epithelial cells (EPCAM, KRT5, KRT8, KRT14, KRT18, KRT19, KLK3, KLK2, NKX3‐1), (e) B cells (CD79A, MS4A1, CD79B, CD19, PAX5, BLNK), (f) Endothelial cells (PECAM1, CDH5, KDR, CD34, VWF, PLVAP, ENG, RAMP2), (g) NK cells (NCAM1, FCGR3A, KLRD1, GNLY, FGFBP2, PRF1, GZMB, KLRF1).

**Figure S3.** Marker gene expression used to identify fibroblasts, mast cells, and myofibroblasts in scRNA‐seq analysis of BPH prostate tissue. Violin plots display expression profiles of canonical marker genes across different cell clusters to validate cell identity. (a) Fibroblasts: COL1A1, LUM, PDGFRA, PTN, DCN, CECL12, IGF1, COL1A2, APOD, FBLN1, MES3. (b) Mast cells: KIT, CPA3, TPSAB1, GATA2, HPGDS. (c) Myofibroblast cells: ACTA2, THY1, RGS5, TAGLN, MYH11, MYH9, FBN2, NDUFA4L2, PPP1R14A, MYL9, PDLIM1

**Figure S4.** Cisplatin induces mRNA expression of senescence markers and SASP cytokines in BPH‐1 and PrEC cells. (a, b) Quantitative RT‐PCR analysis of p16, Rb, IL‐1α, and IL‐6 mRNA expression levels in BPH‐1 (a) and PrEC (b) cells after cisplatin treatment. The results show a significant upregulation of p16, Rb, and pro‐inflammatory SASP factors (IL‐1α and IL‐6) following cisplatin treatment. Data are presented as mean ± SD. Statistical significance: *p < 0.05, **p < 0.01, ***p < 0.001.

**Figure S5.** Functional dissection of CD4^+^ T cell subsets and their roles in senescent epithelial cell clearance. (a) Flow cytometry analysis of immune cell subsets in 13 fresh BPH prostate tissues. Representative gating strategy for identifying CD4^+^ T cells (CD3^+^CD4^+^), CD8^+^ T cells (CD3^+^CD8^+^), and B cells (CD3^−^CD19^+^) within CD45^+^ leukocytes. Quantification revealed CD4^+^ T cells as the predominant subset, followed by CD8^+^ T cells and B cells. (b) Functional characterization of CD4^+^ T cells after PMA/ionomycin stimulation. Five subsets were identified via intracellular staining: Perforin^+^ CD4^+^ CTLs, FOXP3^+^ Tregs, IFN‐γ^+^ Th1, IL‐4^+^ Th2, and IL‐17A^+^ Th17. CD4^+^ CTLs were most abundant (~15.7%), followed by Tregs (~8.3%). (c‐) Co‐culture assays assessing apoptosis of senescent epithelial cells (BPH‐1 and PrEC) with immune cells (effector:target = 50:1). Five groups were tested: (1) senescent cells only, (2) B cells, (3) CD4^+^ T cells, (4) CD4^+^ T cells + CTL inhibition (CMA), and (5) CD4^+^ T cells + Treg inhibition (anti‐CD25). Annexin V/PI staining showed that CD4^+^ T cells significantly increased apoptosis compared to the controls and B cells. Blocking CTLs reduced this effect; inhibiting Tregs enhanced it. Statistical significance: *p < 0.05, **p < 0.01, ***p < 0.001.

**Figure S6.** Identification of distinct cell populations in BPH and control samples. (a) UMAP clustering of single‐cell RNA sequencing data based on unsupervised clustering analysis. Each color represents a distinct cell cluster, labeled numerically. This clustering enables the visualization of heterogeneous cell populations present in both BPH and control samples, highlighting the diversity of cell types and states in the dataset. Epithelial cells (5395) were identified by Krt8, Krt18, Krt4, Krt5, and Epcam, showing their distribution in the tissue. Fibroblasts (5337) were marked by Des and Acta2, overlapping with myofibroblasts (175) that also expressed Acta2. Monocytes and macrophages (2161) were characterized by Cd14, Mafb, C5ar1, Mgl2, Cd163, Itgam, and Flt3. CD4+ T cells (347) expressed Cd4, while CD8+ T cells (403) were marked by Cd8a. Smooth muscle cells (SMCs, 404) and myofibroblasts both expressed Acta2, indicating a possible lineage link. Dendritic cells (175) expressed Xcr1, B cells (285) were marked by Cd79a and Cd3e, mast cells (129) by Kit, and endothelial cells (855) by Pecam1 and Vwf. (b) Sample origin labeling on UMAP plot (BPH vs. control). Cells are colored based on their origin, with BPH and control samples labeled differently. This plot provides insight into the distribution and overlap of cell types between the BPH model and the control group, illustrating cell composition changes associated with testosterone‐induced BPH. (c) Feature plots of marker gene expression for identifying cell types. Each subplot represents the expression pattern of a specific marker gene (e.g., Krt8, Cd14, Acta2), with color intensity indicating expression level across cells in the UMAP space. This panel enables the precise identification of cell types such as epithelial cells, fibroblasts, and various immune cells based on characteristic marker genes. (d) Dot plot of top differentially expressed genes across identified cell types. The size of each dot corresponds to the percentage of cells expressing a given gene, and the color intensity represents the average expression level. This summary plot facilitates comparison of marker gene expression across cell types, emphasizing gene expression profiles that distinguish each cell population. (e) Annotated UMAP plot with cell‐type‐specific identities. Based on marker gene expression profiles, each cluster is annotated with the inferred cell type, such as fibroblasts, CD4+ T cells, endothelial cells, and myofibroblasts. This annotation provides a comprehensive view of the cellular composition within the BPH and control samples, contributing to a better understanding of cell‐type‐specific dynamics and roles in BPH progression.

**Figure S7.** Canonical marker gene expression for epithelial, fibroblast, monocyte/macrophage, and endothelial cell populations in mouse BPH single‐cell RNA sequencing data. (a) Expression profiles of epithelial cell marker genes. Epithelial cells (*n* = 5395) were identified by high expression of Epcam, Krt19, Krt8, Krt5, Krt14, Cldn3, Wfdc2, Cdh1, Lcn2, Tacstd2, S100a14, Mmp7, Col6a2, Acpp, and Scgb3a1. (b) Fibroblast marker gene expression. Fibroblasts (*n* = 5337), the major stromal component, were annotated based on expression of Col1a1, Col1a2, Fbln1, Lum, Ptn, Apod, Pdgfra, Igf1, Cxcl12, Meg3, and Dcn. (c) Marker gene expression for monocytes and macrophages. Monocyte/macrophage cells (*n* = 2161) were identified by high expression of genes such as C1qa, C1qb, C1qc, Adgre1, Csf1r, Mrc1, Itgam, Cd14, Cd68, Siglec1, S100a9, S100a8, Ms4a4a, and Gpr34. (d) Endothelial cell marker gene expression. Endothelial cells (*n* = 855) were defined by expression of Vwf, Pecam1, Cdh5, Plvap, Esam, Egfl7, Aqp1, and Emcn.

**Figure S8.** Canonical marker gene expression for CD8^+^ T cells, CD4^+^ T cells, B cells, smooth muscle cells (SMCs), and myofibroblasts in mouse BPH single‐cell RNA sequencing data. (a) CD8^+^ T cells (*n* = 403) were identified by high expression of Cd3e, Cd8a, Cd8b1, Gzmb, Gzma, Xcl1, Tbx21, Nkg7, Ifng, Eomes, and Prf1. (b) CD4^+^ T cells (*n* = 347) were annotated by Cd3e, Cd4, Foxp3, Ctla4, Il7r, Il17a, Il17f, Cd28, Ccr7, and Tnfrsf4. (c) B cells (*n* = 285) were identified using canonical markers Cd19, Cd79a, Cd79b, Ms4a1, Bank1, Ighm, and Vpreb3.(d) Smooth muscle cells (SMCs, *n* = 404) were defined based on expression of Acta2, Myh11, Cnn1, Rgs5, Tagln, Ppp1r14a, Myl9, and Mylk. (e) Myofibroblasts (*n* = 175) were marked by Acta2, Col1a2, Tagln, Meg3, Igf1, and Myl9.

**Figure S9.** Canonical marker expression of dendritic cells, mast cells, and epithelial subtypes in mouse BPH single‐cell RNA sequencing data. (a) Dendritic cells (*n* = 175) were identified by high expression of Itgax, Xcr1, Flt3, Clec9a, Clec10a, and H2‐Ab1. (b) Mast cells (*n* = 129) were annotated using Tpsab1, Kit, Cpa3, Rgs13, Hpgds, Il1rl1, Gata2, and Mcp1. (c) Epithelial subtypes were further classified based on marker genes: Basal epithelial cells were defined by Krt5, Krt14, and Trp63; luminal epithelial cells by Nkx3‐1; and neuroendocrine epithelial cells by Chga.

**Figure S10.** Immune interactions and senescence‐associated pathways in senescent epithelial cells in a mouse model. (a) GSVA Scores on UMAP: Visualization of the distribution of GSVA scores across various cell subpopulations, presented on a UMAP plot. Each point represents a single cell, colored based on its GSVA score, highlighting significant variations across identified cell types such as epithelial cells, fibroblasts, and monocytes/macrophages. (b) Proportion of cell types above mean GSVA score: Pie chart showing the proportions of cell subpopulations whose GSVA scores are above the mean, with emphasis on the prevalence of senescence‐associated gene expression within epithelial cells, fibroblasts, and monocytes/macrophages. (c) UMAP of senescent vs. non‐senescent epithelial cells: UMAP visualization differentiating senescent from non‐senescent epithelial cells based on their GSVA scores. This plot illustrates the distinct clustering of senescent cells, correlated with enhanced senescence markers. (d) Volcano plot highlighting senescence pathway genes: Differential gene expression analysis results are displayed in a volcano plot, with key senescence‐related genes such as Cdkn1c (p57) marked to show significant upregulation in senescent epithelial cells. (e) GSEA enrichment pathways in senescent epithelial cells: Gene set enrichment analysis results showcasing pathways significantly enriched in senescent epithelial cells. Pathways include immune‐related functions, cell migration, and apoptotic signaling, which support the hypothesis of immune‐mediated clearance of senescent cells. (f) Selected enrichment bubble plot: Visualization of significantly enriched pathways using a bubble plot. Pathways such as cellular senescence, immune chemotaxis, and CD4^+^ T cell activation are shown, with the size of bubbles indicating gene count and color intensity representing the statistical significance of enrichment. (g, k) Cell interaction networks: Network diagrams illustrating comprehensive and intricate interactions among various cell subtypes, highlighting the complex interplay within the tissue microenvironment. (h–m) Detailed cell–cell interaction plots: These plots provide an in‐depth look at the interactions between senescent/non‐senescent epithelial cells and other cell types, emphasizing distinct interaction patterns. (j, n) Interaction strength and frequency comparisons: Bar graphs comparing the interaction strength and frequency between senescent and non‐senescent epithelial cells with CD4+ T cells, underscoring the enhanced capacity of senescent cells to recruit immune cells. (o–q) CXCL pathway interaction networks: Chord and circular diagrams illustrating the CXCL pathway interaction networks among different cell types, highlighting the role of CXCL signaling in mediating cellular interactions. (r) CXCL interaction strength and count: Bar graphs displaying the CXCL‐mediated interaction strength, comparing senescent with non‐senescent epithelial cells in relation to CD4+ T cells. This showcases differential signaling capacity between cell states.

**Figure S11.** Analysis of senescent and non‐senescent epithelial cells in a mouse model of BPH. (a) UMAP plot showing all cell types, with epithelial cells further classified into four subtypes: luminal, basal, epithelial–stromal intermediate, and stem basal cells. (b) Box plot of cellular clusters, colored by cell type, highlighting the GSVA score of BPH groups across different cell subtypes. (c) UMAP plot showing high versus low GSVA score groups within BPH epithelial cells, illustrating the distribution of senescent versus non‐senescent cells. (d) Volcano plot of differentially expressed genes between senescent and non‐senescent BPH epithelial cells, highlighting downregulated Cdk5rap3 and upregulated Cdk1nc, along with genes like Sparc, Igfbp7, Igfbp3, and Thbs1, contributing to a pro‐senescent microenvironment. (e) GSEA plots showing enriched pathways in BPH senescent epithelial cells. The FRIDMAN_SENESCENCE_UP gene set indicates an active senescence program. GOBP_NEGATIVE_REGULATION_OF_MITOTIC_CELL_CYCLE supports cell cycle inhibition, consistent with Cdk5rap3 downregulation and Cdk1nc upregulation. GSE17974_0H_VS_24H_IN_VITRO_ACT_CD4_TCELL_UP suggests recruitment of activated CD4+ T cells, and WU_CELL_MIGRATION implies enhanced immune cell recruitment. (f) Enrichment bubble plot of immune‐related and senescence pathways in BPH senescent epithelial cells. (g) Boxplot comparing GSVA scores for the FRIDMAN_SENESCENCE_UP gene set across individual epithelial cell types, showing elevated scores in BPH subtypes.

**Table S1.** The characteristics of the 31 patients.
**Table S2**. Human and mouse gene list used for cell annotations.
**Table S3**. Differentially expressed genes (DEGs) between senescent and non‐senescent epithelial cells in Human BPH tissues (scRNA‐seq).
**Table S3.1**. Genes mentioned in the main text of senescent and non‐senescent epithelial cells in human BPH tissues (scRNA‐seq).
**Table S4**. Gene set enrichment analysis (GSEA) results based on DEGs between senescent and non‐senescent epithelial cells in human BPH tissues (scRNA‐seq).
**Table S5**. Bubble plot of enriched pathways (MSigDB) based on DEGs between senescent and non‐senescent epithelial cells.
**Table S6**. Differentially expressed genes (DEGs) between senescent and non‐senescent epithelial cells isolated by laser capture microdissection.
**Table S6.1**. Heatmap of FRIDMAN_SENESCENCE_UP genes identified by laser‐captured senescent epithelial cells.
**Table S7**. GSEA results based on DEGs from LCM‐isolated senescent vs. non‐senescent epithelial cells.
**Table S8**. Bubble plot of enriched pathways from DEGs of LCM‐isolated senescent vs. non‐senescent epithelial Cells.
**Table S9**. DEGs between aged and young prostate tissues from the ADEIP database.
**Table S10**. GSEA of DEGs between aged and young prostate tissues (ADEIP).
**Table S11**. Bubble plot of enriched pathways in aged vs. young prostate tissue (ADEIP Dataset).
**Table S12**. CD4^+^ T Cells co‐cultured with senescent vs. non‐senescent epithelial cells—expression matrix.
**Table S13**. GSEA results for CD4^+^ T Cells co‐cultured with senescent vs. non‐senescent epithelial cells.
**Table S14**. Bubble plot of enriched pathways in CD4^+^ T cells co‐cultured with senescent vs. non‐senescent epithelial cells.
**Table S15**. Heatmap of differentially expressed senescence‐ and MHC‐II‐related genes.
**Table S16**. BPH group vs. control group—differentially expressed genes.
**Table S17**. BPH group vs. control group—GSEA results.
**Table S18**. BPH + rmCXCL13 group vs. BPH group—differentially expressed genes.
**Table S19**. BPH + rmCXCL13 group vs. BPH group—GSEA results.
**Table S20**. rmCXCL13‐treated group vs. rmCXCL13 + anti‐CD4‐treated group—differentially expressed genes.
**Table S21**. rmCXCL13‐treated group vs. rmCXCL13 + anti‐CD4‐treated group—GSEA results.
**Table S22**. DEGs between senescent and non‐senescent epithelial cells in mouse scRNA‐seq data.
**Table S23**. GSEA of mouse senescent vs. non‐senescent epithelial DEGs.
**Table S24**. Bubble plot of enriched pathways in senescent vs. non‐senescent mouse epithelial cells (scRNA‐seq).
**Table S25**. DEGs between senescent and non‐senescent epithelial cells in BPH mouse scRNA‐seq data.
**Table S26**. GSEA of DEGs between senescent and non‐senescent epithelial cells in BPH mouse prostate.
**Table S27**. Bubble plot of enriched pathways in senescent vs. non‐senescent epithelial cells in BPH mouse scRNA‐seq.
**Table S28**. Abbreviations.
**Table S29**. Detailed methods.

## Data Availability

All sequencing data supporting this study have been deposited in the NCBI Gene Expression Omnibus (GEO). Publicly available single‐cell RNA‐seq data were retrieved under accession numbers GSE226237, GSM6547516, and GSM6547515. Newly generated datasets are available under GSE297404, GSE297402, and GSE297417. Age‐related gene expression data were obtained from the ADEIP database (http://gepia.cancer‐pku.cn/adeip), which was used for comparative transcriptomic analyses between young and aged prostate tissues.
