# Supplementary figures and images for: Single‐Cell Sequencing Reveals That CD4 + T Cells Eliminate Senescent Prostate Epithelium to Delay Progression of Benign Prostatic Hyperplasia
Source: Aging Cell. 2025 Jul 27;24(10):e70180. doi: 10.1111/acel.70180 (PMC12507426; doi:10.1111/acel.70180)

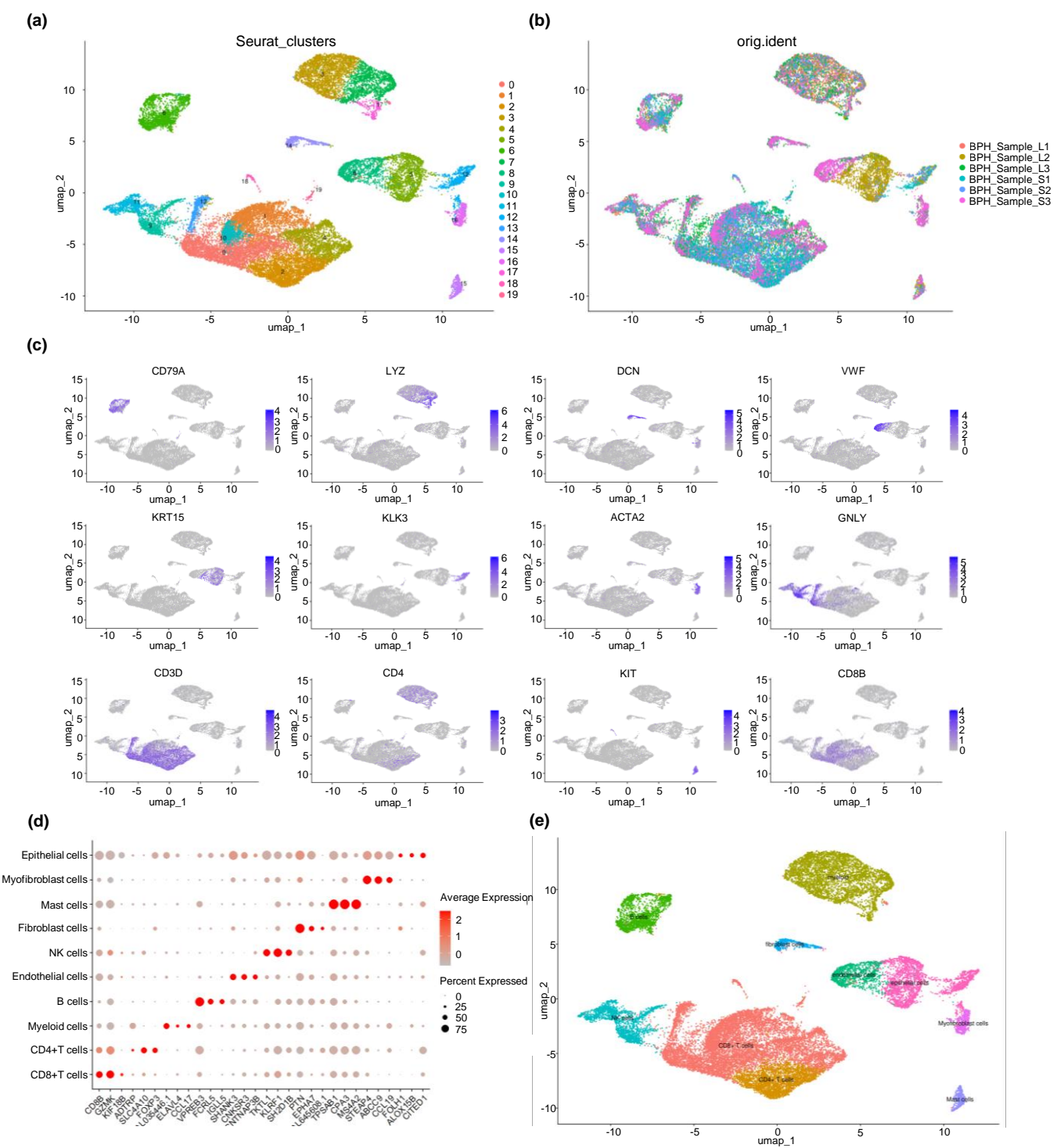

Supplement: Supplementary file 1 — Figure S1. Single‐cell RNA sequencing of prostate tissues from six BPH patients reveals distinct cell populations and functional heterogeneity. (a) UMAP plot showing the clustering of 27,146 cells from six BPH patients into 20 distinct cell populations. Each color represents a unique cluster identified based on transcriptomic similarity. (b) UMAP plot displaying the origin of cells from six individual BPH samples (L1‐S3), indicating the shared and unique cellular distributions across different patients. (c) Feature plots illustrating the expression of key marker genes across the UMAP projections. Marker genes include CD79A (B cells), LYZ (myeloid cells), DCN (fibroblasts), VWF (endothelial cells), KLK3 (epithelial cells), ACTA2 (myofibroblasts), GNLY (NK cells), CD3D (T cells), CD4 (CD4+ T cells), KIT (mast cells), and CD8B (CD8+ T cells), confirming the identity of each cell population. (d) Dot plot summarizing the expression of marker genes across the identified cell types. The size of the dots represents the percentage of cells expressing each gene within the population, and the color intensity represents the average expression level of each marker. (e) UMAP plot highlighting the distinct clustering of major cell types, including epithelial cells, CD8+ T cells, CD4+ T cells, myeloid cells, fibroblasts, mast cells, NK cells, endothelial cells, myofibroblasts, and B cells, indicating functional heterogeneity across these populations in BPH tissues. [file ACEL-24-e70180-s005.pdf]

(a)

CD8+T cells

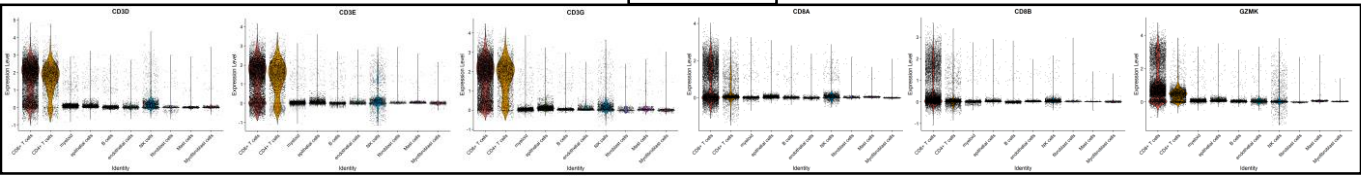

(b)

CD4+T cells

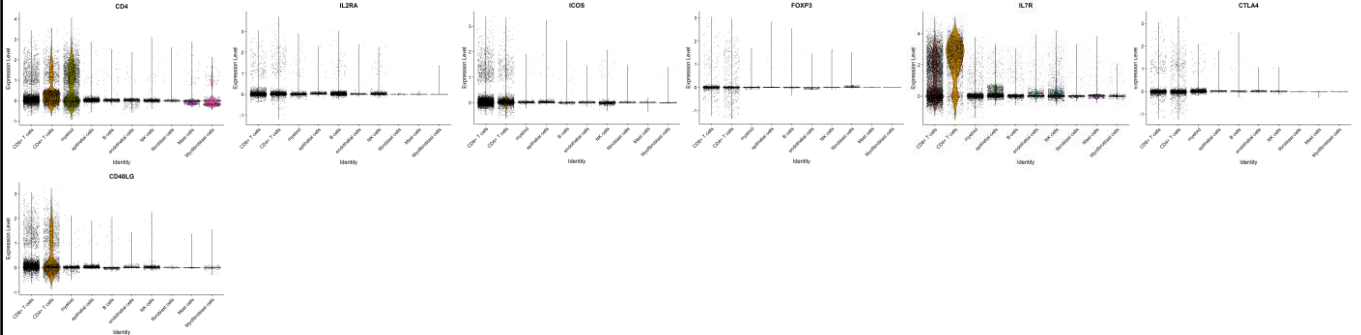

(c)

Myeloid cells

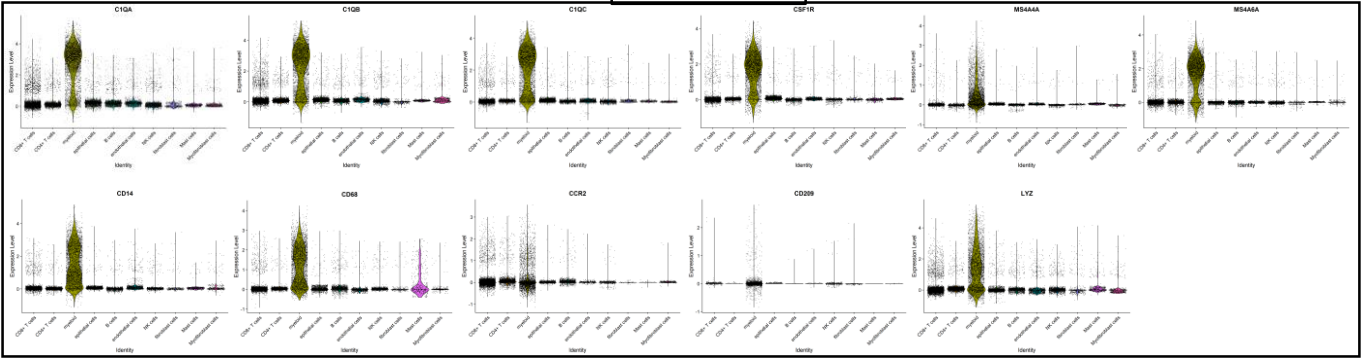

(d)

Epithelial cells

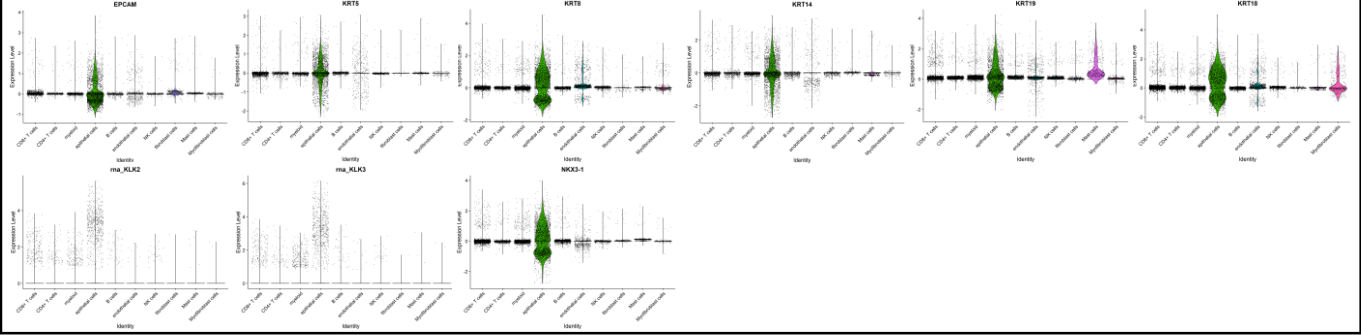

(e)

B cells

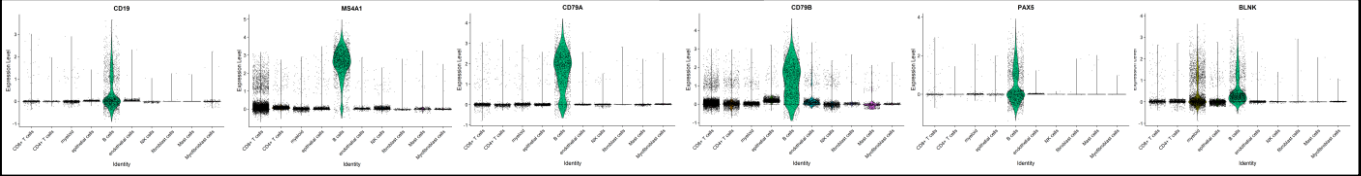

(f)

Endothelial cells

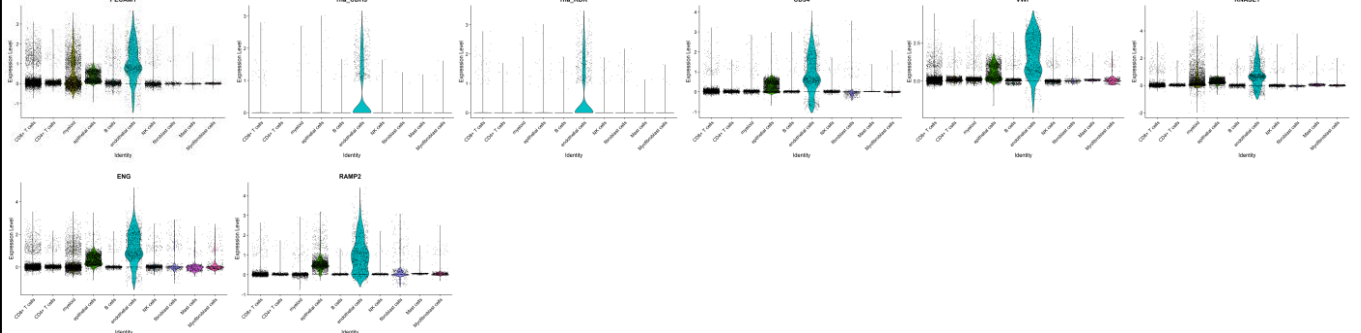

(g)

NK cells

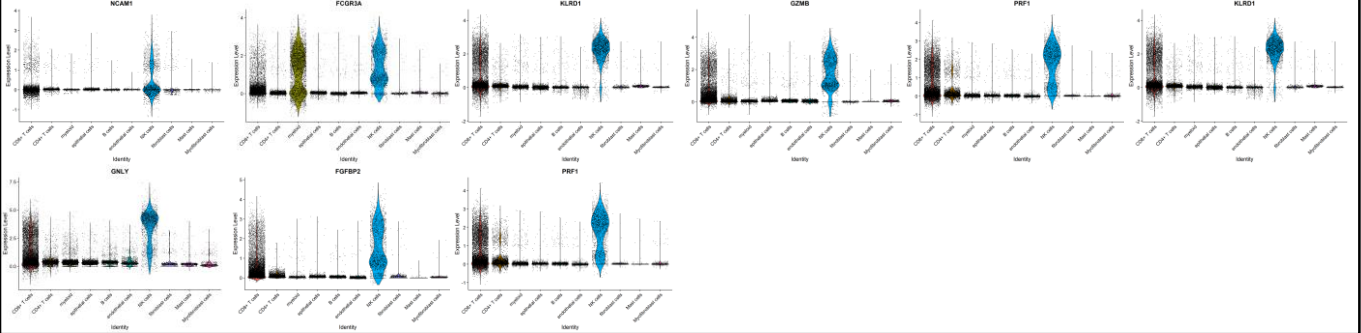

Supplement: Supplementary file 2 — Figure S2. Canonical marker gene expression across major cell types in the human BPH single‐cell dataset. Violin plots showing expression of canonical markers used to annotate the major cell lineages in the integrated scRNA‐seq dataset: (a) CD8+ T cells (CD3D, CD3E, CD3G, CD8A, CD8B, GZMK), (b) CD4+ T cells (CD4, IL2RA, ICOS, FOXP3, IL7R, CTLA4, CD40LG), (c) Myeloid cells (C1QA, C1QB, C1QC, CSF1R, MS4A6A, MS4A4A, CD14, CD68, CCR2, CD209, LYZ), (d) Epithelial cells (EPCAM, KRT5, KRT8, KRT14, KRT18, KRT19, KLK3, KLK2, NKX3‐1), (e) B cells (CD79A, MS4A1, CD79B, CD19, PAX5, BLNK), (f) Endothelial cells (PECAM1, CDH5, KDR, CD34, VWF, PLVAP, ENG, RAMP2), (g) NK cells (NCAM1, FCGR3A, KLRD1, GNLY, FGFBP2, PRF1, GZMB, KLRF1). [file ACEL-24-e70180-s002.pdf]

(a)

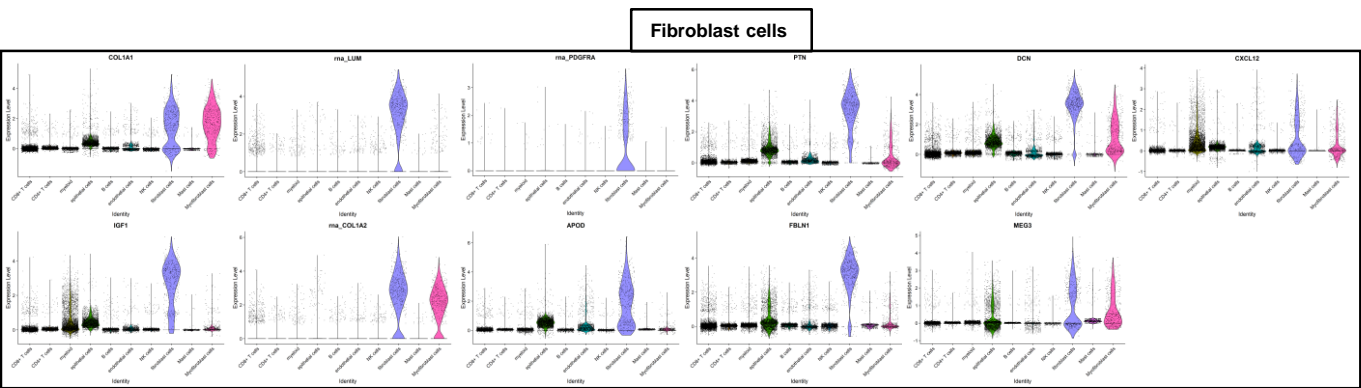

(b)

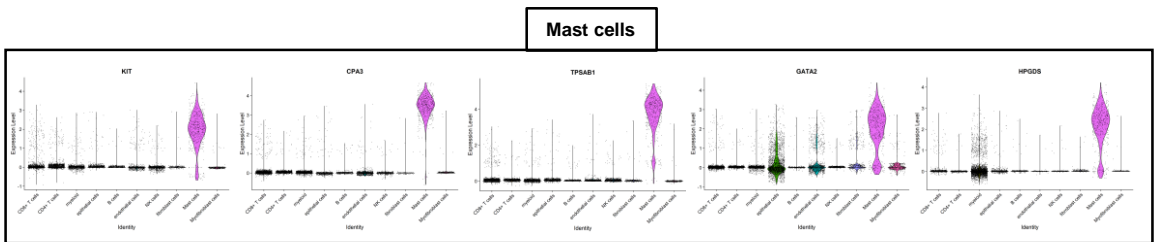

(c)

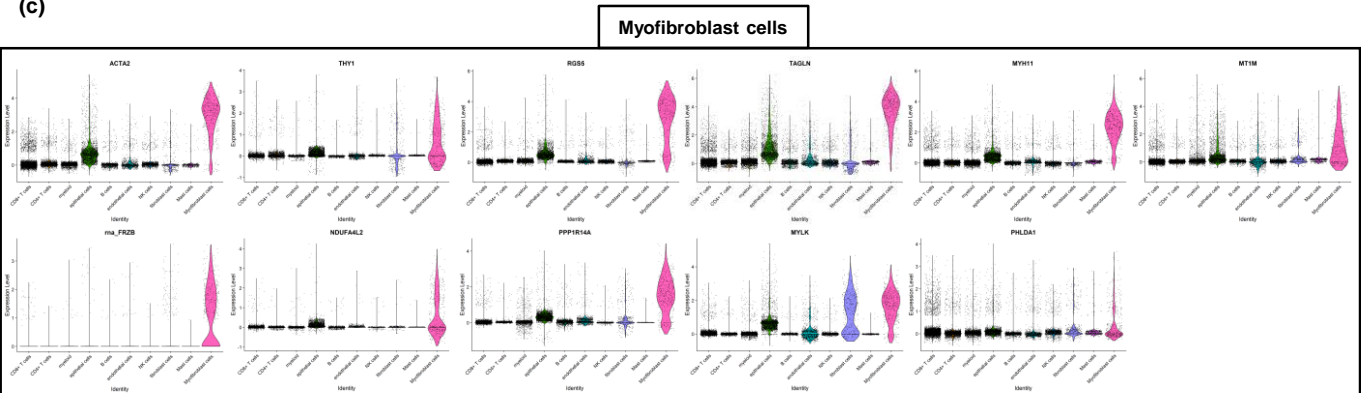

Supplement: Supplementary file 3 — Figure S3. Marker gene expression used to identify fibroblasts, mast cells, and myofibroblasts in scRNA‐seq analysis of BPH prostate tissue. Violin plots display expression profiles of canonical marker genes across different cell clusters to validate cell identity. (a) Fibroblasts: COL1A1, LUM, PDGFRA, PTN, DCN, CECL12, IGF1, COL1A2, APOD, FBLN1, MES3. (b) Mast cells: KIT, CPA3, TPSAB1, GATA2, HPGDS. (c) Myofibroblast cells: ACTA2, THY1, RGS5, TAGLN, MYH11, MYH9, FBN2, NDUFA4L2, PPP1R14A, MYL9, PDLIM1 [file ACEL-24-e70180-s006.pdf]

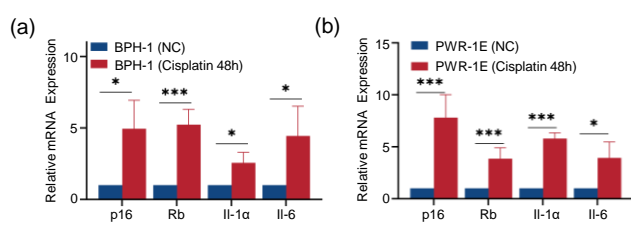

Supplement: Supplementary file 4 — Figure S4. Cisplatin induces mRNA expression of senescence markers and SASP cytokines in BPH‐1 and PrEC cells. (a, b) Quantitative RT‐PCR analysis of p16, Rb, IL‐1α, and IL‐6 mRNA expression levels in BPH‐1 (a) and PrEC (b) cells after cisplatin treatment. The results show a significant upregulation of p16, Rb, and pro‐inflammatory SASP factors (IL‐1α and IL‐6) following cisplatin treatment. Data are presented as mean ± SD. Statistical significance: *p < 0.05, **p < 0.01, ***p < 0.001. [file ACEL-24-e70180-s004.pdf]

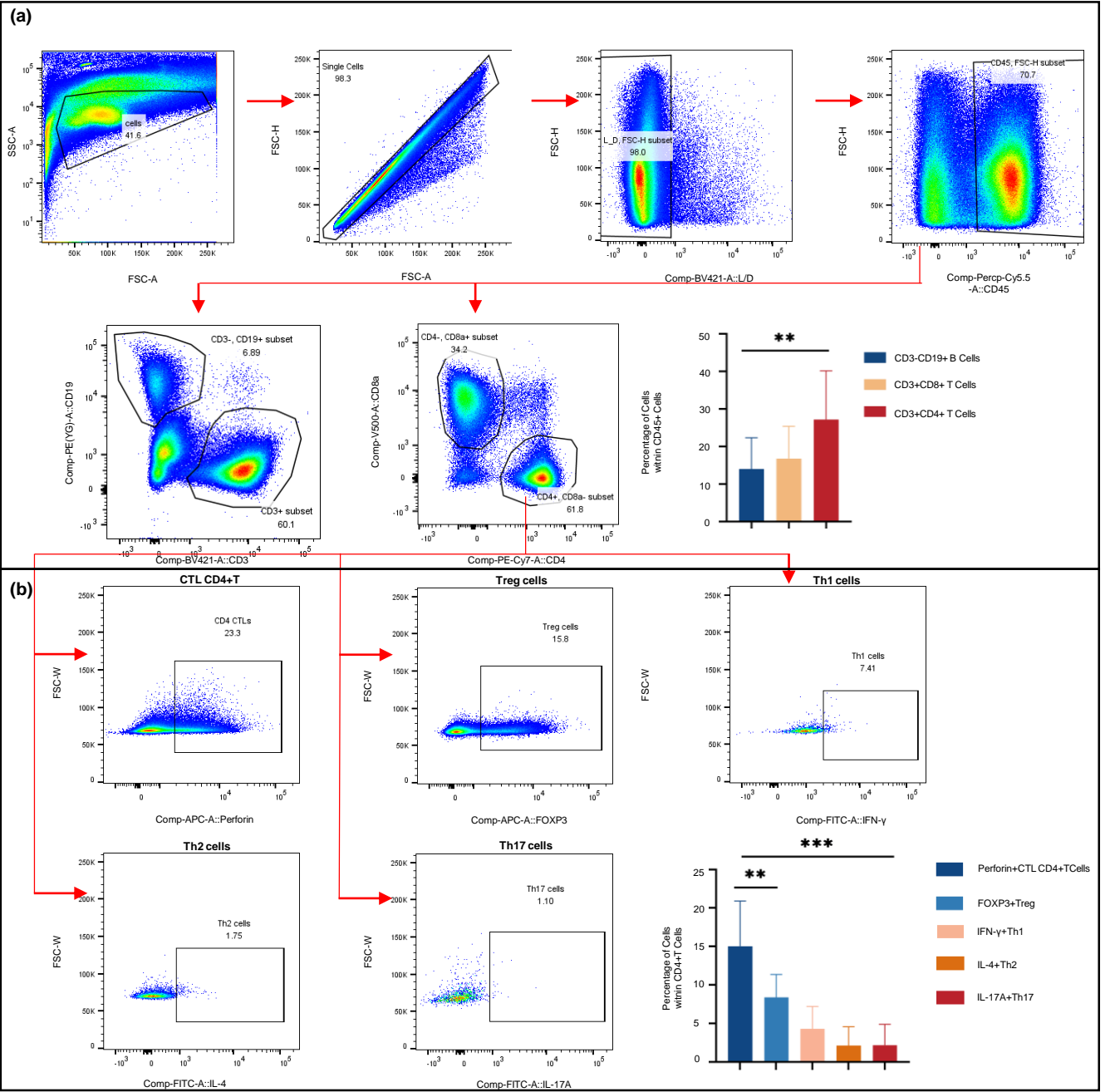

Supplement: Supplementary file 5 — Figure S5. Functional dissection of CD4+ T cell subsets and their roles in senescent epithelial cell clearance. (a) Flow cytometry analysis of immune cell subsets in 13 fresh BPH prostate tissues. Representative gating strategy for identifying CD4+ T cells (CD3+CD4+), CD8+ T cells (CD3+CD8+), and B cells (CD3−CD19+) within CD45+ leukocytes. Quantification revealed CD4+ T cells as the predominant subset, followed by CD8+ T cells and B cells. (b) Functional characterization of CD4+ T cells after PMA/ionomycin stimulation. Five subsets were identified via intracellular staining: Perforin+ CD4+ CTLs, FOXP3+ Tregs, IFN‐γ+ Th1, IL‐4+ Th2, and IL‐17A+ Th17. CD4+ CTLs were most abundant (~15.7%), followed by Tregs (~8.3%). (c‐) Co‐culture assays assessing apoptosis of senescent epithelial cells (BPH‐1 and PrEC) with immune cells (effector:target = 50:1). Five groups were tested: (1) senescent cells only, (2) B cells, (3) CD4+ T cells, (4) CD4+ T cells + CTL inhibition (CMA), and (5) CD4+ T cells + Treg inhibition (anti‐CD25). Annexin V/PI staining showed that CD4+ T cells significantly increased apoptosis compared to the controls and B cells. Blocking CTLs reduced this effect; inhibiting Tregs enhanced it. Statistical significance: *p < 0.05, **p < 0.01, ***p < 0.001. [file ACEL-24-e70180-s001.zip › acel-sup-0005-Supplementaryfigure5a-b.pdf]

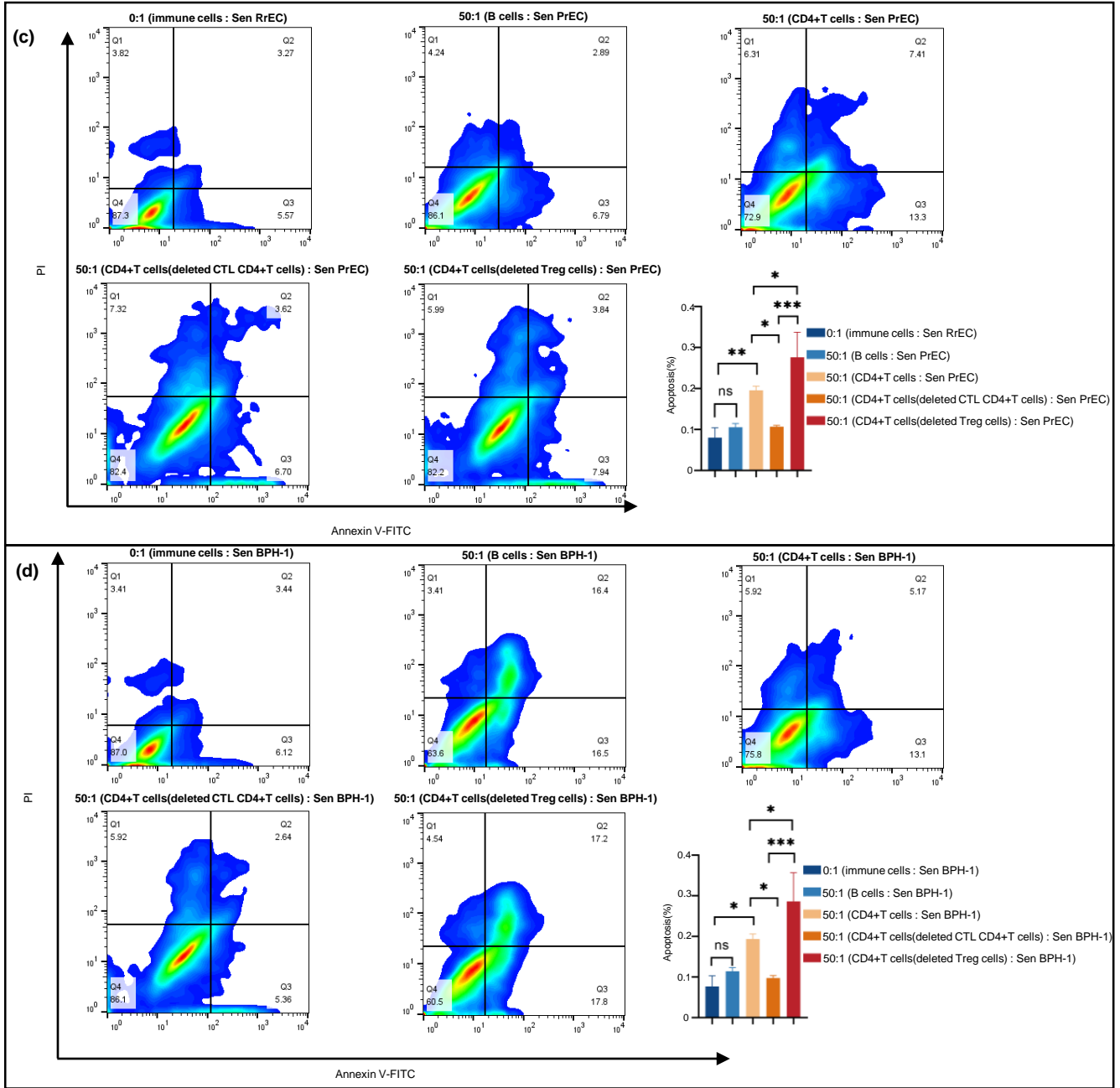

Supplement: Supplementary file 5 — Figure S5. Functional dissection of CD4+ T cell subsets and their roles in senescent epithelial cell clearance. (a) Flow cytometry analysis of immune cell subsets in 13 fresh BPH prostate tissues. Representative gating strategy for identifying CD4+ T cells (CD3+CD4+), CD8+ T cells (CD3+CD8+), and B cells (CD3−CD19+) within CD45+ leukocytes. Quantification revealed CD4+ T cells as the predominant subset, followed by CD8+ T cells and B cells. (b) Functional characterization of CD4+ T cells after PMA/ionomycin stimulation. Five subsets were identified via intracellular staining: Perforin+ CD4+ CTLs, FOXP3+ Tregs, IFN‐γ+ Th1, IL‐4+ Th2, and IL‐17A+ Th17. CD4+ CTLs were most abundant (~15.7%), followed by Tregs (~8.3%). (c‐) Co‐culture assays assessing apoptosis of senescent epithelial cells (BPH‐1 and PrEC) with immune cells (effector:target = 50:1). Five groups were tested: (1) senescent cells only, (2) B cells, (3) CD4+ T cells, (4) CD4+ T cells + CTL inhibition (CMA), and (5) CD4+ T cells + Treg inhibition (anti‐CD25). Annexin V/PI staining showed that CD4+ T cells significantly increased apoptosis compared to the controls and B cells. Blocking CTLs reduced this effect; inhibiting Tregs enhanced it. Statistical significance: *p < 0.05, **p < 0.01, ***p < 0.001. [file ACEL-24-e70180-s001.zip › acel-sup-0006-Supplementaryfigure5c-d.pdf]

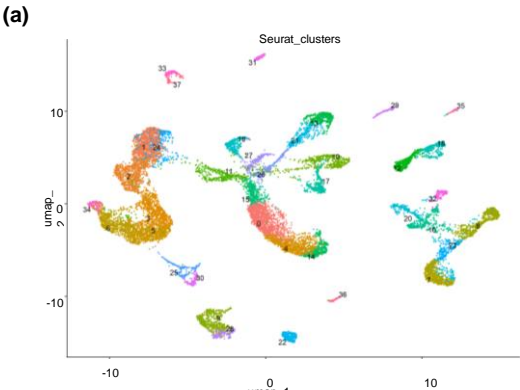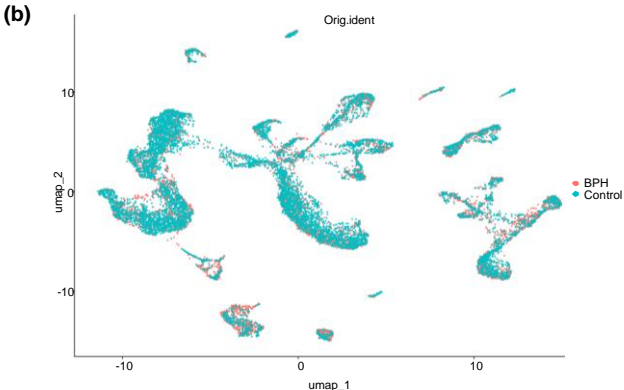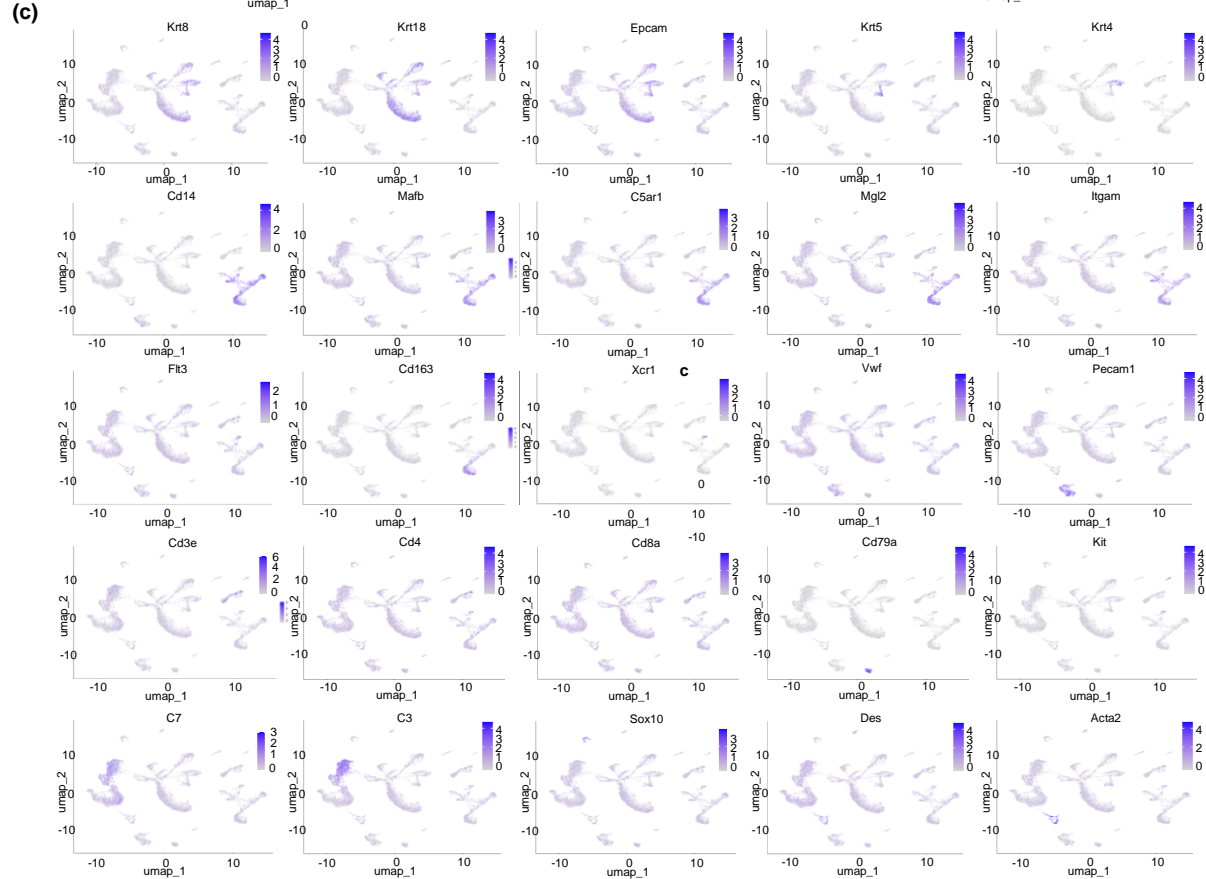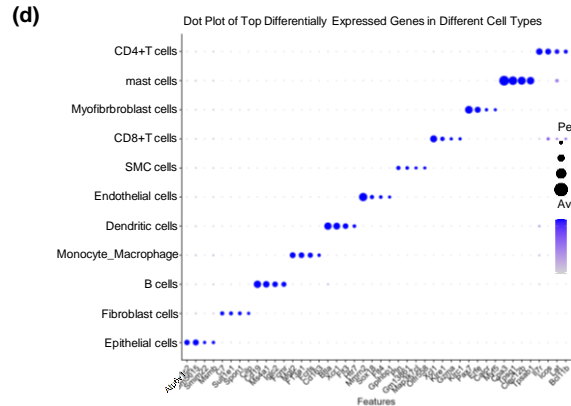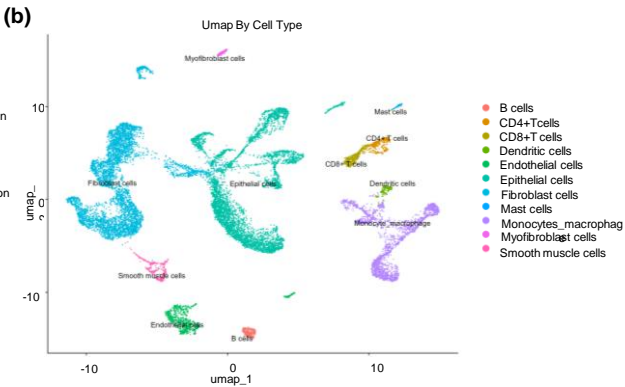

Supplement: Supplementary file 6 — Figure S6. Identification of distinct cell populations in BPH and control samples. (a) UMAP clustering of single‐cell RNA sequencing data based on unsupervised clustering analysis. Each color represents a distinct cell cluster, labeled numerically. This clustering enables the visualization of heterogeneous cell populations present in both BPH and control samples, highlighting the diversity of cell types and states in the dataset. Epithelial cells (5395) were identified by Krt8, Krt18, Krt4, Krt5, and Epcam, showing their distribution in the tissue. Fibroblasts (5337) were marked by Des and Acta2, overlapping with myofibroblasts (175) that also expressed Acta2. Monocytes and macrophages (2161) were characterized by Cd14, Mafb, C5ar1, Mgl2, Cd163, Itgam, and Flt3. CD4+ T cells (347) expressed Cd4, while CD8+ T cells (403) were marked by Cd8a. Smooth muscle cells (SMCs, 404) and myofibroblasts both expressed Acta2, indicating a possible lineage link. Dendritic cells (175) expressed Xcr1, B cells (285) were marked by Cd79a and Cd3e, mast cells (129) by Kit, and endothelial cells (855) by Pecam1 and Vwf. (b) Sample origin labeling on UMAP plot (BPH vs. control). Cells are colored based on their origin, with BPH and control samples labeled differently. This plot provides insight into the distribution and overlap of cell types between the BPH model and the control group, illustrating cell composition changes associated with testosterone‐induced BPH. (c) Feature plots of marker gene expression for identifying cell types. Each subplot represents the expression pattern of a specific marker gene (e.g., Krt8, Cd14, Acta2), with color intensity indicating expression level across cells in the UMAP space. This panel enables the precise identification of cell types such as epithelial cells, fibroblasts, and various immune cells based on characteristic marker genes. (d) Dot plot of top differentially expressed genes across identified cell types. The size of each dot corresponds to t [file ACEL-24-e70180-s007.pdf]

(a)

Epithelial

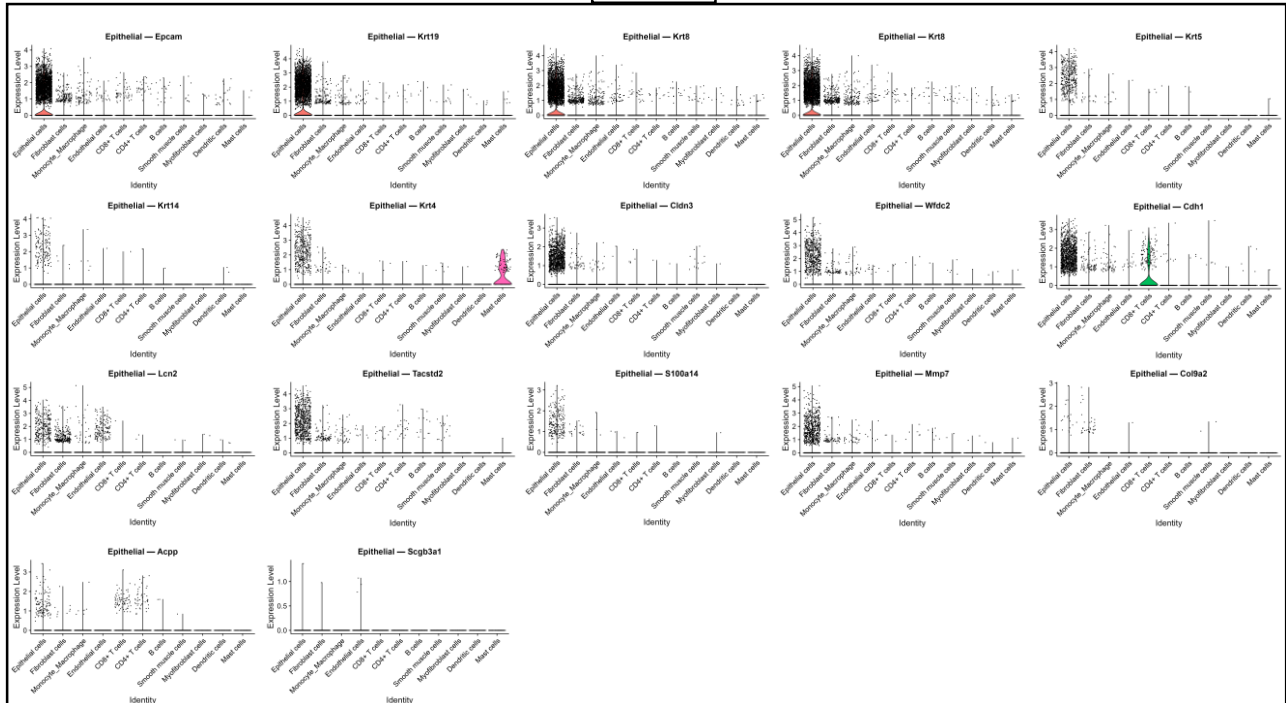

(b)

Fibroblast

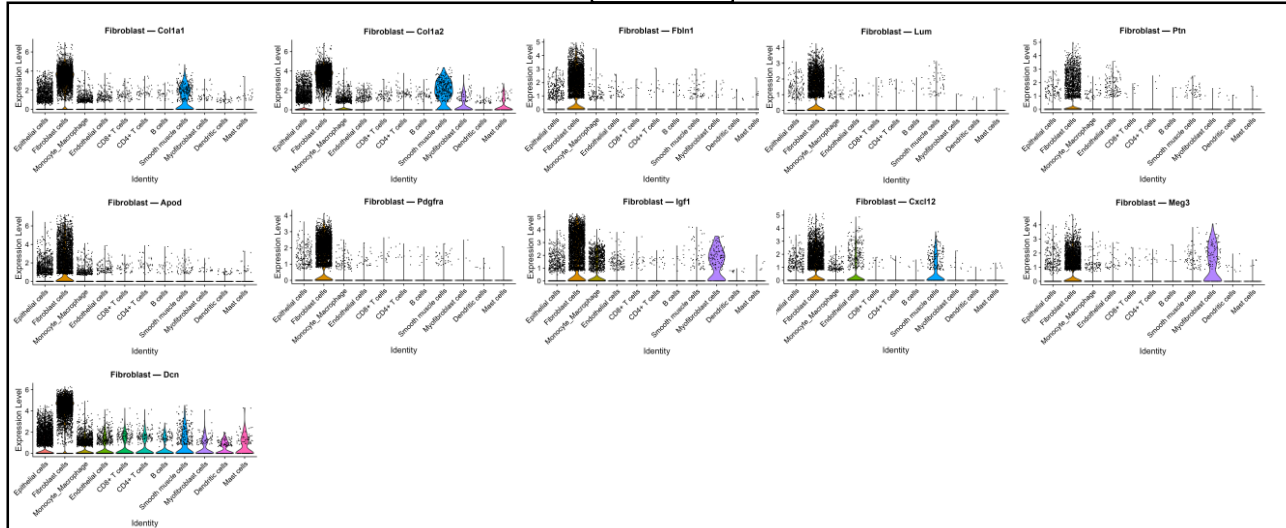

Supplement: Supplementary file 7 — Figure S7. Canonical marker gene expression for epithelial, fibroblast, monocyte/macrophage, and endothelial cell populations in mouse BPH single‐cell RNA sequencing data. (a) Expression profiles of epithelial cell marker genes. Epithelial cells (n = 5395) were identified by high expression of Epcam, Krt19, Krt8, Krt5, Krt14, Cldn3, Wfdc2, Cdh1, Lcn2, Tacstd2, S100a14, Mmp7, Col6a2, Acpp, and Scgb3a1. (b) Fibroblast marker gene expression. Fibroblasts (n = 5337), the major stromal component, were annotated based on expression of Col1a1, Col1a2, Fbln1, Lum, Ptn, Apod, Pdgfra, Igf1, Cxcl12, Meg3, and Dcn. (c) Marker gene expression for monocytes and macrophages. Monocyte/macrophage cells (n = 2161) were identified by high expression of genes such as C1qa, C1qb, C1qc, Adgre1, Csf1r, Mrc1, Itgam, Cd14, Cd68, Siglec1, S100a9, S100a8, Ms4a4a, and Gpr34. (d) Endothelial cell marker gene expression. Endothelial cells (n = 855) were defined by expression of Vwf, Pecam1, Cdh5, Plvap, Esam, Egfl7, Aqp1, and Emcn. [file ACEL-24-e70180-s012.zip › acel-sup-0008-Supplementaryfigure7a-b.pdf]

(c)

Monocyte\_Macrophage

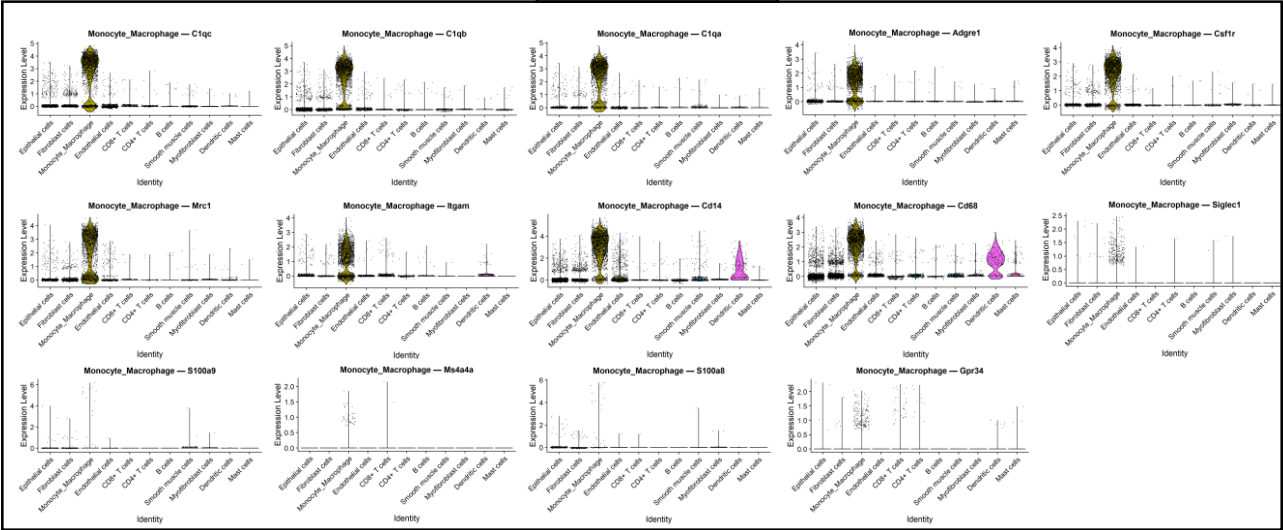

(d)

Endothelial

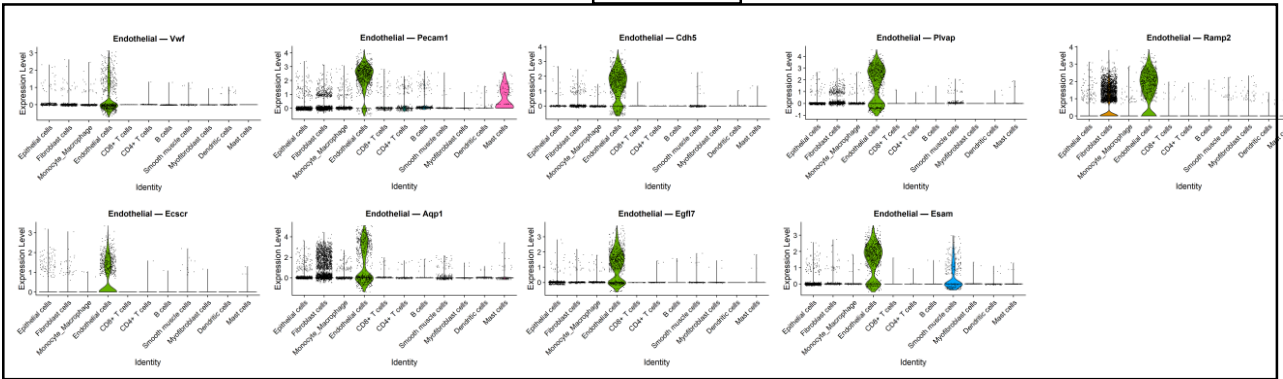

Supplement: Supplementary file 7 — Figure S7. Canonical marker gene expression for epithelial, fibroblast, monocyte/macrophage, and endothelial cell populations in mouse BPH single‐cell RNA sequencing data. (a) Expression profiles of epithelial cell marker genes. Epithelial cells (n = 5395) were identified by high expression of Epcam, Krt19, Krt8, Krt5, Krt14, Cldn3, Wfdc2, Cdh1, Lcn2, Tacstd2, S100a14, Mmp7, Col6a2, Acpp, and Scgb3a1. (b) Fibroblast marker gene expression. Fibroblasts (n = 5337), the major stromal component, were annotated based on expression of Col1a1, Col1a2, Fbln1, Lum, Ptn, Apod, Pdgfra, Igf1, Cxcl12, Meg3, and Dcn. (c) Marker gene expression for monocytes and macrophages. Monocyte/macrophage cells (n = 2161) were identified by high expression of genes such as C1qa, C1qb, C1qc, Adgre1, Csf1r, Mrc1, Itgam, Cd14, Cd68, Siglec1, S100a9, S100a8, Ms4a4a, and Gpr34. (d) Endothelial cell marker gene expression. Endothelial cells (n = 855) were defined by expression of Vwf, Pecam1, Cdh5, Plvap, Esam, Egfl7, Aqp1, and Emcn. [file ACEL-24-e70180-s012.zip › acel-sup-0009-Supplementaryfigure7c-d.pdf]

(a)

CD8+T cells

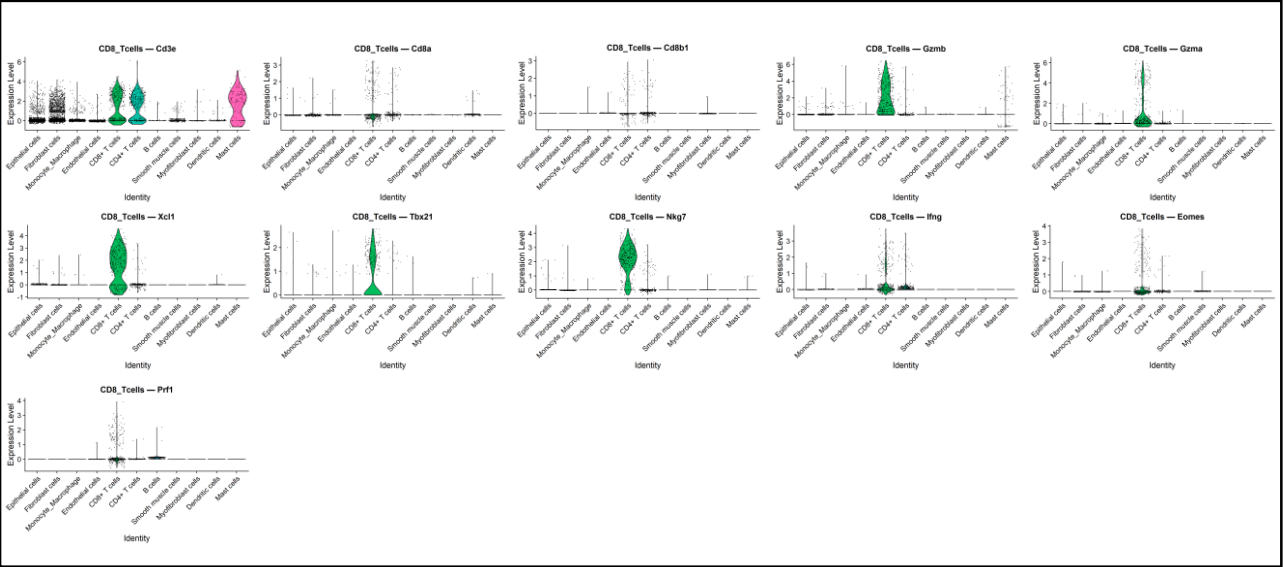

(b)

CD4+T cells

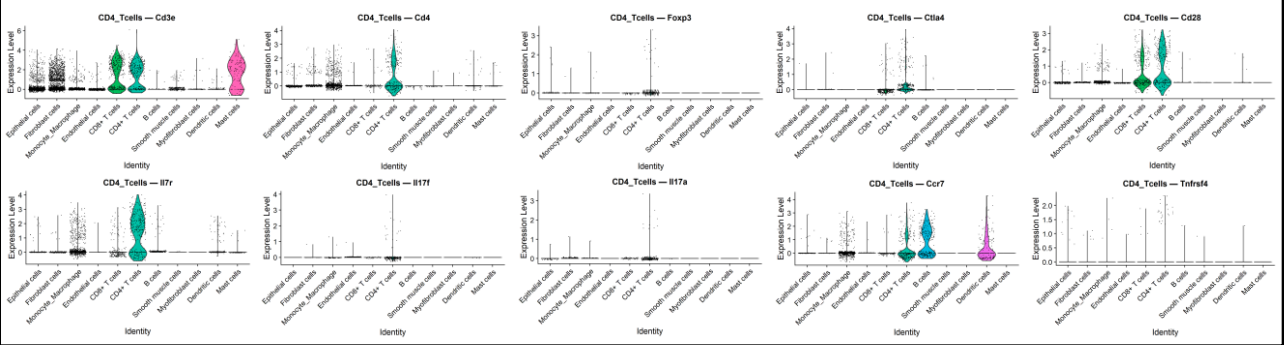

Supplement: Supplementary file 8 — Figure S8. Canonical marker gene expression for CD8+ T cells, CD4+ T cells, B cells, smooth muscle cells (SMCs), and myofibroblasts in mouse BPH single‐cell RNA sequencing data. (a) CD8+ T cells (n = 403) were identified by high expression of Cd3e, Cd8a, Cd8b1, Gzmb, Gzma, Xcl1, Tbx21, Nkg7, Ifng, Eomes, and Prf1. (b) CD4+ T cells (n = 347) were annotated by Cd3e, Cd4, Foxp3, Ctla4, Il7r, Il17a, Il17f, Cd28, Ccr7, and Tnfrsf4. (c) B cells (n = 285) were identified using canonical markers Cd19, Cd79a, Cd79b, Ms4a1, Bank1, Ighm, and Vpreb3.(d) Smooth muscle cells (SMCs, n = 404) were defined based on expression of Acta2, Myh11, Cnn1, Rgs5, Tagln, Ppp1r14a, Myl9, and Mylk. (e) Myofibroblasts (n = 175) were marked by Acta2, Col1a2, Tagln, Meg3, Igf1, and Myl9. [file ACEL-24-e70180-s003.zip › Supplementary Figure 8a-b.pdf]

(c)

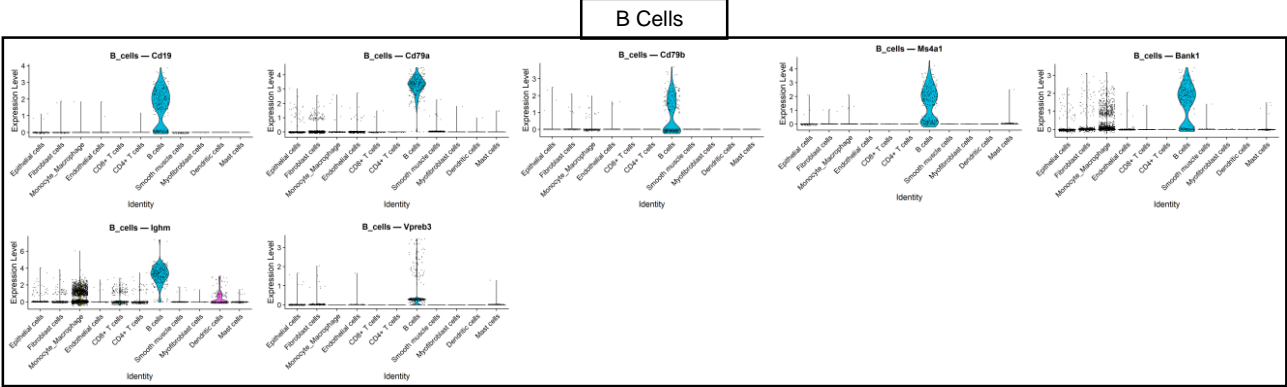

(d)

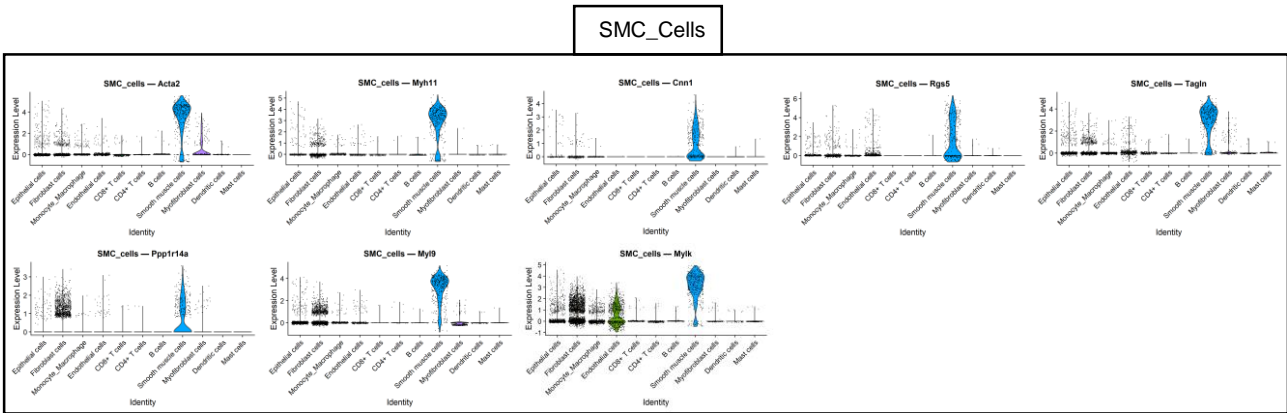

(e)

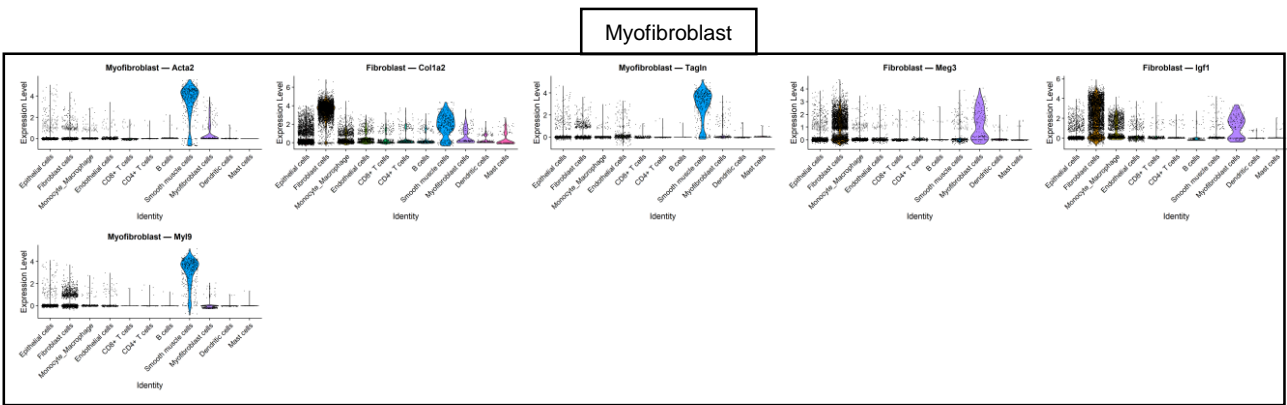

Supplement: Supplementary file 8 — Figure S8. Canonical marker gene expression for CD8+ T cells, CD4+ T cells, B cells, smooth muscle cells (SMCs), and myofibroblasts in mouse BPH single‐cell RNA sequencing data. (a) CD8+ T cells (n = 403) were identified by high expression of Cd3e, Cd8a, Cd8b1, Gzmb, Gzma, Xcl1, Tbx21, Nkg7, Ifng, Eomes, and Prf1. (b) CD4+ T cells (n = 347) were annotated by Cd3e, Cd4, Foxp3, Ctla4, Il7r, Il17a, Il17f, Cd28, Ccr7, and Tnfrsf4. (c) B cells (n = 285) were identified using canonical markers Cd19, Cd79a, Cd79b, Ms4a1, Bank1, Ighm, and Vpreb3.(d) Smooth muscle cells (SMCs, n = 404) were defined based on expression of Acta2, Myh11, Cnn1, Rgs5, Tagln, Ppp1r14a, Myl9, and Mylk. (e) Myofibroblasts (n = 175) were marked by Acta2, Col1a2, Tagln, Meg3, Igf1, and Myl9. [file ACEL-24-e70180-s003.zip › Supplementary Figure 8c-d.pdf]

(a)

Dendritic cells

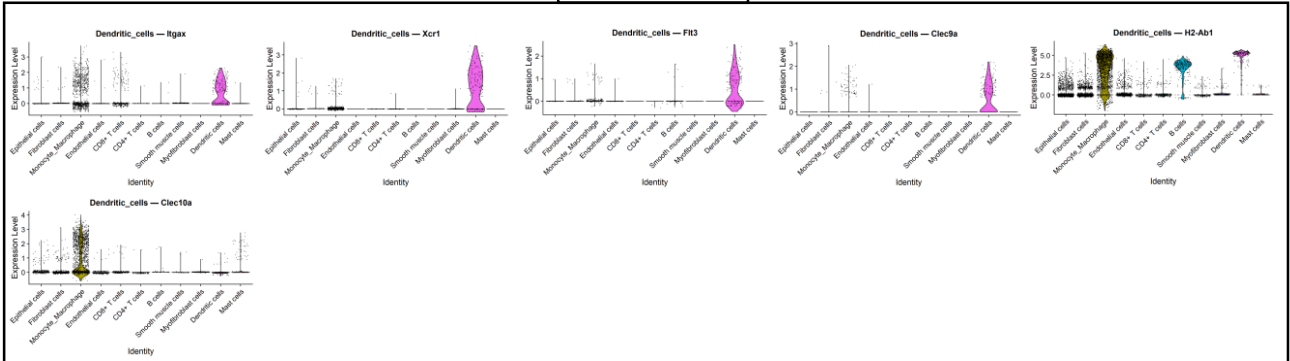

(b)

Mast cells

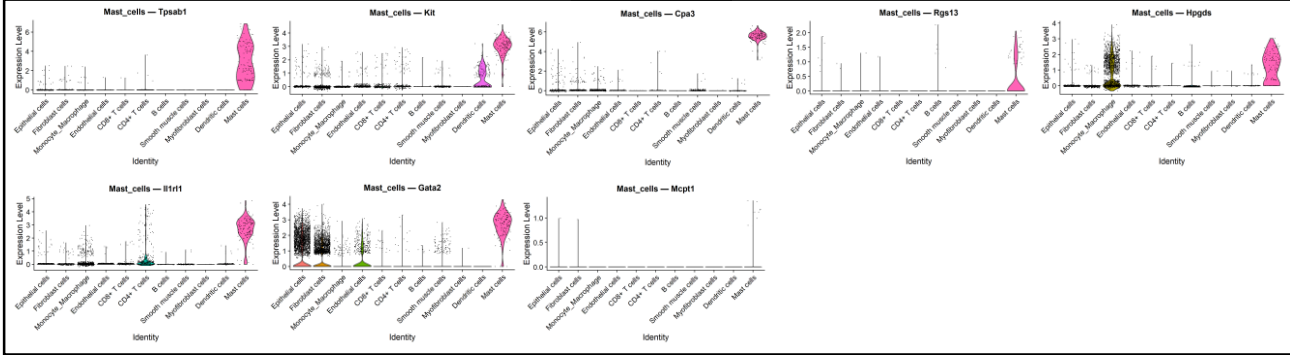

(c)

Epithelial subtypes

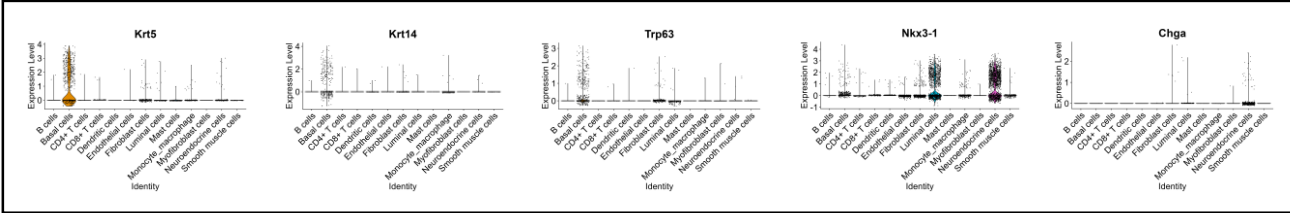

Supplement: Supplementary file 9 — Figure S9. Canonical marker expression of dendritic cells, mast cells, and epithelial subtypes in mouse BPH single‐cell RNA sequencing data. (a) Dendritic cells (n = 175) were identified by high expression of Itgax, Xcr1, Flt3, Clec9a, Clec10a, and H2‐Ab1. (b) Mast cells (n = 129) were annotated using Tpsab1, Kit, Cpa3, Rgs13, Hpgds, Il1rl1, Gata2, and Mcp1. (c) Epithelial subtypes were further classified based on marker genes: Basal epithelial cells were defined by Krt5, Krt14, and Trp63; luminal epithelial cells by Nkx3‐1; and neuroendocrine epithelial cells by Chga. [file ACEL-24-e70180-s008.pdf]

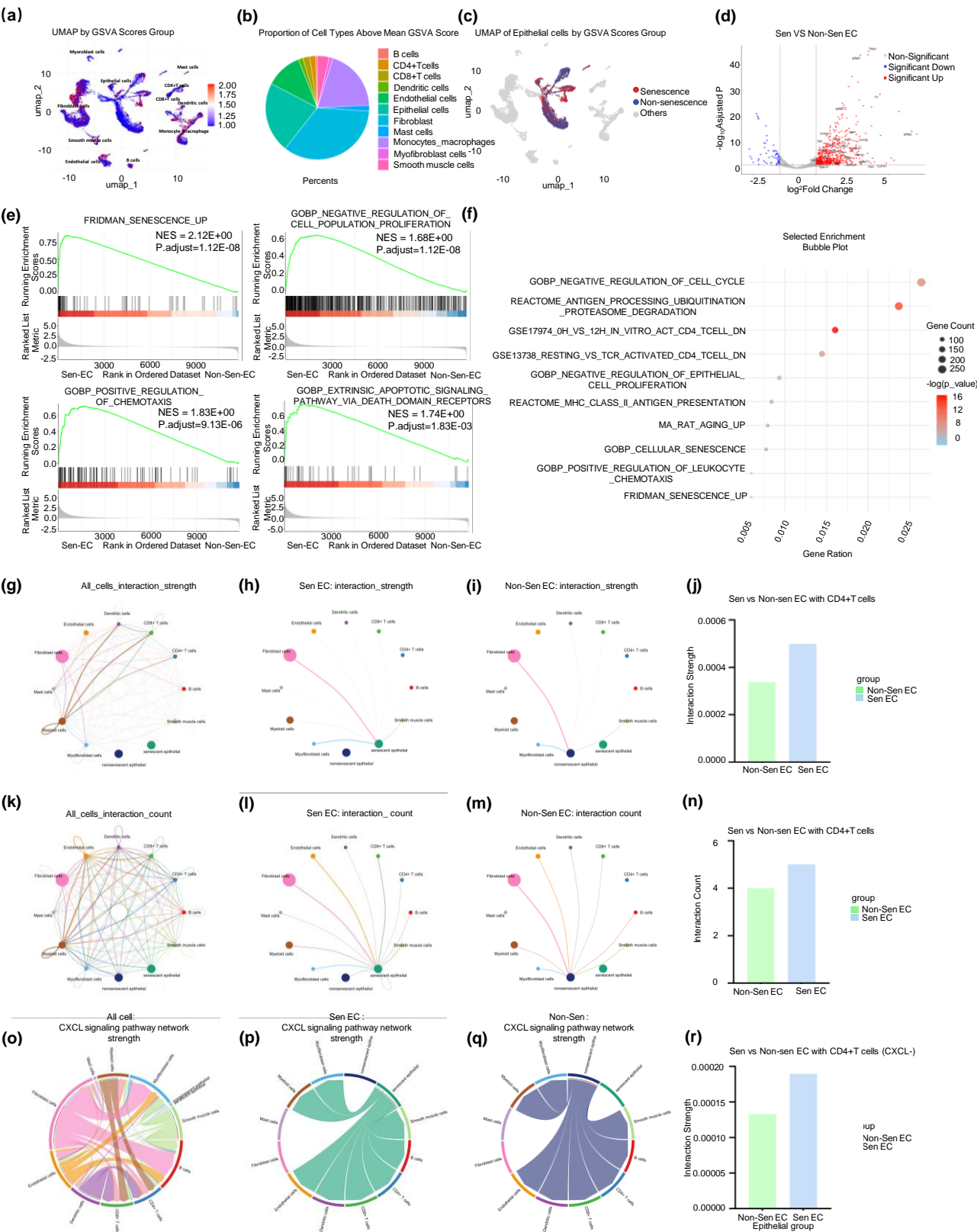

Supplement: Supplementary file 10 — Figure S10. Immune interactions and senescence‐associated pathways in senescent epithelial cells in a mouse model. (a) GSVA Scores on UMAP: Visualization of the distribution of GSVA scores across various cell subpopulations, presented on a UMAP plot. Each point represents a single cell, colored based on its GSVA score, highlighting significant variations across identified cell types such as epithelial cells, fibroblasts, and monocytes/macrophages. (b) Proportion of cell types above mean GSVA score: Pie chart showing the proportions of cell subpopulations whose GSVA scores are above the mean, with emphasis on the prevalence of senescence‐associated gene expression within epithelial cells, fibroblasts, and monocytes/macrophages. (c) UMAP of senescent vs. non‐senescent epithelial cells: UMAP visualization differentiating senescent from non‐senescent epithelial cells based on their GSVA scores. This plot illustrates the distinct clustering of senescent cells, correlated with enhanced senescence markers. (d) Volcano plot highlighting senescence pathway genes: Differential gene expression analysis results are displayed in a volcano plot, with key senescence‐related genes such as Cdkn1c (p57) marked to show significant upregulation in senescent epithelial cells. (e) GSEA enrichment pathways in senescent epithelial cells: Gene set enrichment analysis results showcasing pathways significantly enriched in senescent epithelial cells. Pathways include immune‐related functions, cell migration, and apoptotic signaling, which support the hypothesis of immune‐mediated clearance of senescent cells. (f) Selected enrichment bubble plot: Visualization of significantly enriched pathways using a bubble plot. Pathways such as cellular senescence, immune chemotaxis, and CD4+ T cell activation are shown, with the size of bubbles indicating gene count and color intensity representing the statistical significance of enrichment. (g, k) Cell interaction networks: Network diagrams illustrating c [file ACEL-24-e70180-s009.pdf]

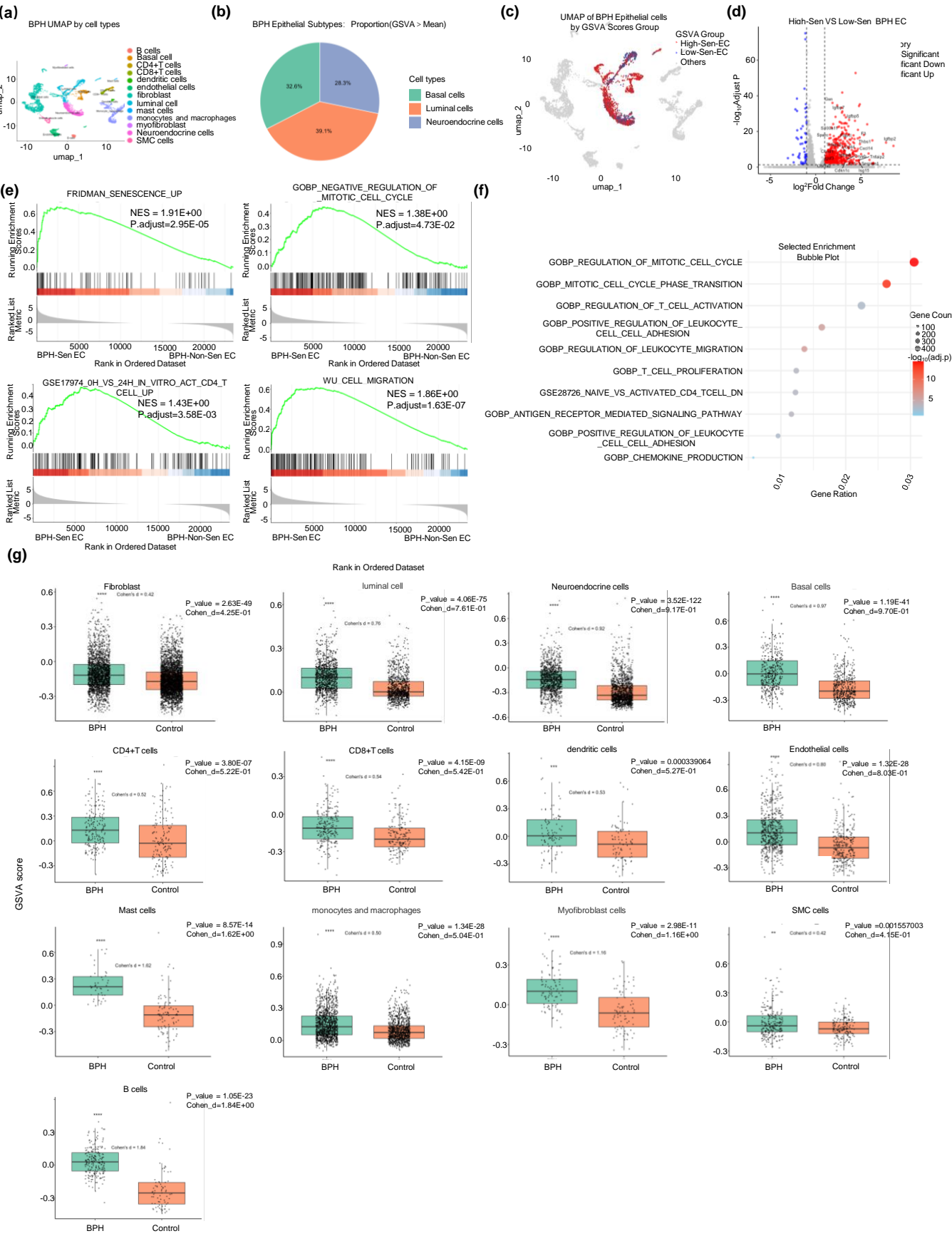

Supplement: Supplementary file 11 — Figure S11. Analysis of senescent and non‐senescent epithelial cells in a mouse model of BPH. (a) UMAP plot showing all cell types, with epithelial cells further classified into four subtypes: luminal, basal, epithelial–stromal intermediate, and stem basal cells. (b) Box plot of cellular clusters, colored by cell type, highlighting the GSVA score of BPH groups across different cell subtypes. (c) UMAP plot showing high versus low GSVA score groups within BPH epithelial cells, illustrating the distribution of senescent versus non‐senescent cells. (d) Volcano plot of differentially expressed genes between senescent and non‐senescent BPH epithelial cells, highlighting downregulated Cdk5rap3 and upregulated Cdk1nc, along with genes like Sparc, Igfbp7, Igfbp3, and Thbs1, contributing to a pro‐senescent microenvironment. (e) GSEA plots showing enriched pathways in BPH senescent epithelial cells. The FRIDMAN_SENESCENCE_UP gene set indicates an active senescence program. GOBP_NEGATIVE_REGULATION_OF_MITOTIC_CELL_CYCLE supports cell cycle inhibition, consistent with Cdk5rap3 downregulation and Cdk1nc upregulation. GSE17974_0H_VS_24H_IN_VITRO_ACT_CD4_TCELL_UP suggests recruitment of activated CD4+ T cells, and WU_CELL_MIGRATION implies enhanced immune cell recruitment. (f) Enrichment bubble plot of immune‐related and senescence pathways in BPH senescent epithelial cells. (g) Boxplot comparing GSVA scores for the FRIDMAN_SENESCENCE_UP gene set across individual epithelial cell types, showing elevated scores in BPH subtypes. [file ACEL-24-e70180-s010.pdf]
