# Supplementary material for: Single‐Cell Sequencing Reveals That CD4 + T Cells Eliminate Senescent Prostate Epithelium to Delay Progression of Benign Prostatic Hyperplasia
Source: Aging Cell. 2025 Jul 27;24(10):e70180. doi: 10.1111/acel.70180 (PMC12507426; doi:10.1111/acel.70180)
Supplement: Supplementary file 12 — Table S1. The characteristics of the 31 patients. Table S2. Human and mouse gene list used for cell annotations. Table S3. Differentially expressed genes (DEGs) between senescent and non‐senescent epithelial cells in Human BPH tissues (scRNA‐seq). Table S3.1. Genes mentioned in the main text of senescent and non‐senescent epithelial cells in human BPH tissues (scRNA‐seq). Table S4. Gene set enrichment analysis (GSEA) results based on DEGs between senescent and non‐senescent epithelial cells in human BPH tissues (scRNA‐seq). Table S5. Bubble plot of enriched pathways (MSigDB) based on DEGs between senescent and non‐senescent epithelial cells. Table S6. Differentially expressed genes (DEGs) between senescent and non‐senescent epithelial cells isolated by laser capture microdissection. Table S6.1. Heatmap of FRIDMAN_SENESCENCE_UP genes identified by laser‐captured senescent epithelial cells. Table S7. GSEA results based on DEGs from LCM‐isolated senescent vs. non‐senescent epithelial cells. Table S8. Bubble plot of enriched pathways from DEGs of LCM‐isolated senescent vs. non‐senescent epithelial Cells. Table S9. DEGs between aged and young prostate tissues from the ADEIP database. Table S10. GSEA of DEGs between aged and young prostate tissues (ADEIP). Table S11. Bubble plot of enriched pathways in aged vs. young prostate tissue (ADEIP Dataset). Table S12. CD4+ T Cells co‐cultured with senescent vs. non‐senescent epithelial cells—expression matrix. Table S13. GSEA results for CD4+ T Cells co‐cultured with senescent vs. non‐senescent epithelial cells. Table S14. Bubble plot of enriched pathways in CD4+ T cells co‐cultured with senescent vs. non‐senescent epithelial cells. Table S15. Heatmap of differentially expressed senescence‐ and MHC‐II‐related genes. Table S16. BPH group vs. control group—differentially expressed genes. Table S17. BPH group vs. control group—GSEA results. Table S18. BPH + rmCXCL13 group vs. BPH group—differentially expressed genes. Table S19. BPH [file ACEL-24-e70180-s011.zip › Supplementary Tables 3.1–29..docx]

**Supplementary Information**

The following supplementary tables are provided in a single Dropbox folder:
🔗 <https://www.dropbox.com/scl/fo/xjkqfwzn5ilashzeimftn/AIxAYGxwzCT27mpbvAvy8IU?rlkey=glh5fcxzjoh43oiigysef9tjt&st=fybfdk7v&dl=0>

**List of Supplementary Tables:**

- **Supplementary Table 3.1**: Genes mentioned in the main text of Senescent and Non-Senescent Epithelial Cells in Human BPH Tissues (scRNA-seq)
- **Supplementary Table 3**: Differentially Expressed Genes (DEGs) Between Senescent and Non-Senescent Epithelial Cells in Human BPH Tissues (scRNA-seq)
- **Supplementary Table 4**: Gene Set Enrichment Analysis (GSEA) Results Based on DEGs Between Senescent and Non-Senescent Epithelial Cells in Human BPH Tissues (scRNA-seq)
- **Supplementary Table 5**: Bubble Plot of Enriched Pathways (MSigDB) Based on DEGs Between Senescent and Non-Senescent Epithelial Cells
- **Supplementary Table 6.1**: Heatmap of FRIDMAN_SENESCENCE_UP Genes Identified by Laser-Captured Senescent Epithelial Cells
- **Supplementary Table 6**: Differentially Expressed Genes (DEGs) Between Senescent and Non-Senescent Epithelial Cells Isolated by Laser Capture Microdissection
- **Supplementary Table 7**: GSEA Results Based on DEGs From LCM-Isolated Senescent vs. Non-Senescent Epithelial Cells
- **Supplementary Table 8**: Bubble Plot of Enriched Pathways From DEGs of LCM-Isolated Senescent vs. Non-Senescent Epithelial Cells
- **Supplementary Table 9**: DEGs Between Aged and Young Prostate Tissues From the ADEIP Database
- **Supplementary Table 10**: GSEA of DEGs Between Aged and Young Prostate Tissues (ADEIP)
- **Supplementary Table 11**: Bubble Plot of Enriched Pathways in Aged vs. Young Prostate Tissue (ADEIP Dataset)
- **Supplementary Table 12**: CD4⁺ T Cells Co-cultured With Senescent vs. Non-Senescent Epithelial Cells – expression matrix
- **Supplementary Table 13**: GSEA Results for CD4⁺ T Cells Co-cultured With Senescent vs. Non-Senescent Epithelial Cells
- **Supplementary Table 14**: Bubble Plot of Enriched Pathways in CD4⁺ T Cells Co-cultured With Senescent vs. Non-Senescent Epithelial Cells
- **Supplementary Table 15**: Heatmap of Differentially Expressed Senescence- and MHC-II–Related Genes
- **Supplementary Table 16**: BPH group vs. Control group – Differentially Expressed Genes
- **Supplementary Table 17**: BPH group vs. Control group – GSEA Results
- **Supplementary Table 18**: BPH + rmCXCL13 group vs. BPH group – Differentially Expressed Genes
- **Supplementary Table 19**: BPH + rmCXCL13 group vs. BPH group – GSEA Results
- **Supplementary Table 20**: rmCXCL13-treated group vs. rmCXCL13 + anti-CD4-treated group – Differentially Expressed Genes
- **Supplementary Table 21**: rmCXCL13-treated group vs. rmCXCL13 + anti-CD4-treated group – GSEA Results
- **Supplementary Table 22**: DEGs Between Senescent and Non-Senescent Epithelial Cells in Mouse scRNA-seq Data
- **Supplementary Table 23**: GSEA of Mouse Senescent vs. Non-Senescent Epithelial DEGs
- **Supplementary Table 24**: Bubble Plot of Enriched Pathways in Senescent vs. Non-Senescent Mouse Epithelial Cells (scRNA-seq)
- **Supplementary Table 25**: DEGs Between Senescent and Non-Senescent Epithelial Cells in BPH Mouse scRNA-seq Data
- **Supplementary Table 26**: GSEA of DEGs Between Senescent and Non-Senescent Epithelial Cells in BPH Mouse Prostate
- **Supplementary Table 27**: Bubble Plot of Enriched Pathways in Senescent vs. Non-Senescent Epithelial Cells in BPH Mouse scRNA-seq
- **Supplementary Table 28**: Abbreviations
- **Supplementary 29**: Detailed Methods
- **Note:** All tables are accessible via the Dropbox link above. If any issues arise when accessing the folder, please contact the corresponding author.
